# Metabolic Cardiomyopathies and Cardiac Defects in Inherited Disorders of Carbohydrate Metabolism: A Systematic Review

**DOI:** 10.3390/ijms24108632

**Published:** 2023-05-11

**Authors:** Federica Conte, Juda-El Sam, Dirk J. Lefeber, Robert Passier

**Affiliations:** 1 Department of Neurology, Donders Institute for Brain, Cognition and Behavior, Radboud University Medical Center, 6525 GA Nijmegen, The Netherlands; 2 Department of Applied Stem Cell Technologies, TechMed Centre, University of Twente, 7522 NH Enschede, The Netherlands; 3 Translational Metabolic Laboratory, Department of Laboratory Medicine, Radboud Institute for Molecular Life Sciences, Radboud University Medical Center, 6525 GA Nijmegen, The Netherlands; 4 Department of Anatomy and Embryology, Leiden University Medical Center, 2333 ZA Leiden, The Netherlands

**Keywords:** heart failure, cardiomyopathies, arrhythmogenic disorders, congenital heart disease, inborn errors of metabolism, disorders of sugar transporters, glycogen storage disorders, disorders of pentose phosphate pathway, lysosomal storage disorders, congenital disorders of glycosylation

## Abstract

Heart failure (HF) is a progressive chronic disease that remains a primary cause of death worldwide, affecting over 64 million patients. HF can be caused by cardiomyopathies and congenital cardiac defects with monogenic etiology. The number of genes and monogenic disorders linked to development of cardiac defects is constantly growing and includes inherited metabolic disorders (IMDs). Several IMDs affecting various metabolic pathways have been reported presenting cardiomyopathies and cardiac defects. Considering the pivotal role of sugar metabolism in cardiac tissue, including energy production, nucleic acid synthesis and glycosylation, it is not surprising that an increasing number of IMDs linked to carbohydrate metabolism are described with cardiac manifestations. In this systematic review, we offer a comprehensive overview of IMDs linked to carbohydrate metabolism presenting that present with cardiomyopathies, arrhythmogenic disorders and/or structural cardiac defects. We identified 58 IMDs presenting with cardiac complications: 3 defects of sugar/sugar-linked transporters (GLUT3, GLUT10, THTR1); 2 disorders of the pentose phosphate pathway (G6PDH, TALDO); 9 diseases of glycogen metabolism (GAA, GBE1, GDE, GYG1, GYS1, LAMP2, RBCK1, PRKAG2, G6PT1); 29 congenital disorders of glycosylation (ALG3, ALG6, ALG9, ALG12, ATP6V1A, ATP6V1E1, B3GALTL, B3GAT3, COG1, COG7, DOLK, DPM3, FKRP, FKTN, GMPPB, MPDU1, NPL, PGM1, PIGA, PIGL, PIGN, PIGO, PIGT, PIGV, PMM2, POMT1, POMT2, SRD5A3, XYLT2); 15 carbohydrate-linked lysosomal storage diseases (CTSA, GBA1, GLA, GLB1, HEXB, IDUA, IDS, SGSH, NAGLU, HGSNAT, GNS, GALNS, ARSB, GUSB, ARSK). With this systematic review we aim to raise awareness about the cardiac presentations in carbohydrate-linked IMDs and draw attention to carbohydrate-linked pathogenic mechanisms that may underlie cardiac complications.

## 1. Introduction

Heart failure (HF) is a chronic, progressive condition in which heart functionality becomes heavily compromised and cannot sustain the oxygen demands of the body, ultimately resulting in death. Several studies have established that 40–50% of patients with HF die within 5 years from diagnosis [1]. It has been estimated to affect over 64 million people worldwide and the societal burden has been estimated at around $108 billion per year globally, representing an unacceptably high medical and societal burden [2].

The pathogenesis of HF is extremely complex, heterogeneous and multifactorial. HF can result from environmental factors (e.g., lifestyle), genetic predisposition, hereditary disorders or a combination of these three. Nonetheless, the most common causes of HF are cardiomyopathies (CMs) and congenital cardiac defects [1].

CMs represent a clinically heterogeneous group of disorders resulting in abnormal heart structure and functionality [3]. They are conventionally divided into familial (genetic, inherited) and non-familial (acquired) forms [4]. During the past three decades, a number of disease-causing genes of different CMs have been identified and further subdivided CMs based on their poly-, oligo- or mono-genic origin [3].

A relevant portion of genetic cardiomyopathies are monogenic. Considering the two most frequent forms of CMs, it has been estimated that over 60% of hypertrophic cardiomyopathies (HCMs) and 30–35% of dilated cardiomyopathies (DCMs) have monogenic etiology [5].

Among the CMs and structural heart defects arising from monogenic diseases, the number of those linked to genetic defects affecting proteins and enzymes linked to cellular metabolism has been steadily growing. Genetic disorders of the metabolism represent their own class of rare diseases, often referred to as inborn metabolic disorders (IMDs), or inborn errors of metabolism. To date, over 1000 IMDs have been described. They can be caused by inherited or de novo genetic mutations that disrupt one or more pathways of the cellular metabolism [6,7]. Although IMDs are very rare, taken collectively they affect 1 in 2500 births and account for 0.4% of all child deaths worldwide [8,9]. Cardiac manifestations in IMDs are largely variable in pattern and severity but encompass CMs, cardiac structural defects, arrhythmogenic disorders and can be the cause of HF [10]. Cardiac manifestations have been linked to genetic alteration of several pathways of cellular metabolism, including fatty acid oxidation, mitochondrial respiration, ion transporters and, relevant to the scope of this review, carbohydrate metabolism [10].

Carbohydrates play a central role in the physiology, development and metabolism of cardiac muscle tissue, or myocardium. Glucose, galactose, fructose and other sugars can serve as substrates for different metabolic pathways linked to cardiac tissue development, metabolism and functionality. First, carbohydrates contribute to the energy production in cardiac cells. Specifically, sugars represent the second most important energy source for the heart, covering 30–40% of the cardiac energy demand in the adult myocardium. Via glycolysis, glucose is transformed anaerobically into pyruvate while producing a net gain of two molecules of adenosine-5′-triphosphate (ATP). ATP provides readily releasable energy, stored in the bond between the second and third phosphate groups, to myosin heads in the contractile machinery of cardiac cells. The hydrolysis of ATP allows myosin to attach to actin filaments, starting a new contractile cycle, whose reiteration enables the pumping function of the myocardium. Second, carbohydrates can be used to store energy in the form of glycogen. Glycogen is a branched polymer of glucose molecules that constitutes an endogenous metabolic reserve able to grant up to 16 kilojoules (KJ) of energy per gram (g) oxidized, via the release of glucose into glycolysis [11]. Third, carbohydrates contribute to the biosynthesis of nucleotides and thus of DNA and RNA, by providing ribose 5-phosphate. The synthesis of this building block is enabled by the so called pentose phosphate pathway (PPP), which also produces nicotinamide adenine dinucleotide phosphate (NADPH) to use in reductive biosynthesis, such as that of fatty acids [12]. Lastly, carbohydrates can modulate protein and lipid maturation by fueling the synthesis of nucleotide sugars, such as UDP–glucose, UDP–galactose and GDP–mannose, which represent the precursors of essential post-translational modifications such as glycosylation and O-GlcNAcylation [13]. Glycosylation consists in the attachment of oligosaccharide chains, called glycans, to acceptor molecules, such as proteins and lipids, to enable their final configuration and functionality. In the context of the heart, glycoconjugates are mostly important for signal transduction, depolarization, and cell adhesion. O-GlcNAcylation, by contrast, indicates the process of attachment of *N*-acetylglucosamine onto serine or threonine residues of proteins and is responsible for the modulation of intracellular proteins, such as transcriptional factors [13].

This brief overview only roughly outlines the main key roles played by carbohydrates in the development and functionality of cardiac tissue. However, the knowledge of the exact molecular mechanisms by which these metabolic alterations generate cardiac defects remain mostly elusive and often understudied. The availability of more advanced cardiological clinical assays and the growing awareness of metabolic diseases have further widened the list of IMDs with cardiac involvement, but clinical information from clinical literature and case reports remains fragmented.

In this review, we offer a systematic and comprehensive overview of the different classes of monogenic carbohydrate-linked IMDs that present with cardiomyopathies, arrhythmogenic disorders and/or cardiac defects.

## 2. Method and Search Strategies

### 2.1. Definition of the Search Terms

**Figure 1:**
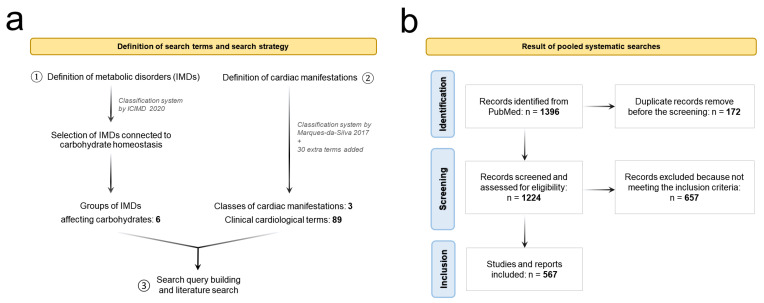
PRISMA flow diagram of the systematic search strategy. (**a**) Definition of the terms used for the systematic search performed for each IMD [7,14]. (**b**) Pooled results of the systematic searches, including the total number of studies found, screened and included. The terms used to build the search queries are reported in Supplementary Table S1. The details of the separate systematic searches performed for each disorder (e.g., excluded and included articles) are reported in Supplementary Tables S2 and S3. For four disorders, G6PDH-, GAA-. GLA- and GALNS-deficiency, respectively, we selected the articles manually and not systematically due to the high volume of records identified (>300 articles/each).

To set up our systematic literature search, we needed to define the categorization systems for IMDs and for cardiac manifestations. Concerning IMDs, despite the lack of universal consensus when it comes to systematic classification, we chose the recently proposed International Classification of Inherited Metabolic Disorders (ICIMD) [7]. Of the 1450 disorders described by the ICIMD, we identified six categories of IMDs connected to carbohydrate metabolism and homeostasis: (1) disorders of sugar transporters; (2) disorders of fructose metabolism; (3) disorders of the pentose phosphate pathway; (4) disorders affecting glycogen metabolism; (5) disorders of glycosylation and galactose metabolism; (6) lysosomal storage diseases linked to carbohydrate homeostasis (Figure 1a).

Likewise, for the classification of the cardiac manifestations, we used as a base the system proposed in Marques-da-Silva et al. [14], which we further expanded by the addition of 33 extra terms. In total, our system includes 89 different cardiological clinical manifestations, divided into three categories: (1) arrhythmogenic disorders, (2) cardiomyopathies, and (3) structural cardiac and valvular defects (Figure 1a, Table 1).

### 2.2. Systematic Literature Search

A comprehensive literature search based on PRISMA guidelines [15] was conducted in February 2023 using PubMed (MeSH term-based search and free text search), in concert with the online databases Inborn Errors of Metabolism Knowledgebase (IEMbase) and Online Mendelian Inheritance in Man (OMIM) (Figure 1).

Each disorder included in the selected IMD groups was investigated in the databases mentioned hereinabove to identify articles and case reports reporting IMD patients displaying cardiac symptoms. The details on the search query building are reported in Supplementary Table S1. Articles were included only when presenting genetic diagnosis of the IMDs and clinical details on the cardiac manifestations in patients. Articles reporting experimental findings from animal models or in vitro experiments which did not mention any cardiac clinical data in patients were excluded. Articles not written in English were also excluded.

In cases where the same patients were mentioned across multiple studies, we counted them only once each to avoid bias in the estimation of the affected patients, but all linked articles were nevertheless reported.

## 3. Results

Our systematic search produced 567 included articles, which led to 58 IMDs reported with cardiac manifestations in patients (Figure 1), divided into 5 classes: 3 disorders affecting cytosolic sugar-linked transporters, 2 disorders of the pentose phosphate pathway, 9 diseases of glycogen metabolism, 29 congenital disorders of glycosylation and galactose metabolism and 15 carbohydrate-linked lysosomal storage diseases. For one of the selected carbohydrate-linked IMD groups, namely the disorders of fructose metabolism, no reports of patients displaying cardiac manifestations have been found.

### 3.1. Disorders of Plasma Membrane Transporters of Sugars and Linked Metabolites

Monosaccharides such as glucose, galactose and mannose are neutral hydrophilic molecules and cannot easily navigate through the lipophilic bilayer of cell membranes. Transport of monosaccharides, especially glucose, is the rate-limiting step in carbohydrate metabolism and energy homeostasis. Besides anchored proteins that function as hydrophilic pores that enable passive exchange between cytoplasm and ECM (unspecific transcellular transport), monosaccharides, can be absorbed by the cell via specialized transporters. Three classes of eukaryotic sugar transporters have been characterized, namely glucose transporters (GLUTs), sodium–glucose symporters (SGLTs) and the most recently discovered family, SWEETs [16]. SGLTs are sodium–sugar symporters, meaning that they transport glucose and other monosaccharides against their concentration gradient. Conversely, GLUTs and SWEETs are uniporters, which facilitate sugar transport along the sugar gradient. Interestingly, SGLT1 and SGLT2 have been associated with the development of diabetes-linked cardiac symptoms and SGLT2 inhibitors have been trialed in recent years as a preventive treatment for HF and other cardiovascular complications [17,18,19,20]. In the context of congenital metabolic disorders, out of the six disorders affecting carbohydrate transporters reported to date, three present with cardiac manifestations [21] (Table 2).

#### 3.1.1. GLUT3 Deficiency

Mutations affecting the solute carrier family 2 member 3 (*SLC2A3*) gene, located on chromosome 12p13.31, have been linked to congenital syndromic heart defects, although the molecular mechanisms still remain elusive [22]. *SLC2A3* encodes glucose transporter 3 (GLUT3), a membrane uniporter with a high affinity for glucose, which is predominantly expressed in the brain, pharyngeal arches and heart (highest expression detected in the left ventricular outflow tract during development) [22,23]. So far, a limited number of clinical reports have been published, but Ma et al. [22] reported 14 patients with SLC2A3 mutation, of which 6 presented with secundum ASD, right heart enlargement with pulmonary hypertension, TC and multi-hole PVSD, and one patient died due to heart disease (Table 2, Supplementary Tables S2 and S3).

#### 3.1.2. GLUT10 Deficiency

Arterial tortuosity syndrome (ATORS) [OMIM:208050] is caused by an AR mutation in the *SLC2A10* gene at location 20q13.12, resulting in GLUT10 deficiency. GLUT10 is critical for cardiovascular development by facilitating both TGFβ signaling and mitochondrial respiration [24]. We found 24 patients affected by ATORS and with confirmed genetic diagnoses of *SLC2A10* mutations [25,26,27,28,29]. ATORS is characterized by tortuosity of the aorta and middle-sized arteries, elongation, stenosis and aneurysm formation in major arteries, leading to disrupted elastic fibers in the medial layers of arterial walls [24,25]. Other symptoms include joint laxity and defects of the connective tissue and one patient also reported stomach displacement and bilateral hip dislocation [26]. In our review, we identified four patients with ATORS presenting alongside cardiac symptoms, including cardiac murmur, severe kinking of the aortic arch and arteries, aggressive DAR and progressive RDA, MVP, CM, ICM and PPS [28,29,30] (Table 2, Supplementary Tables S2 and S3).

#### 3.1.3. THTR1 Deficiency

Thiamine-responsive megaloblastic anemia (TRMA) syndrome [OMIM:249270] is an AR disorder of the thiamine (also known as vitamin B1) transporter. It is caused by homozygous mutations of the *SLC19A2* gene on location 1q24.2, which encodes thiamine transporter type 1 (THTR1). Thiamine is important for the oxidation of carbohydrates, especially glucose. Upon its conversion to thiamine–pyrophosphate, thiamine acts as a coenzyme for three key enzymes involved in carbohydrate metabolism: transketolase (TK) in the pentose phosphate pathway (PPP), pyruvate dehydrogenase (PDH) in the terminal part of glycolysis and α-ketoglutarate dehydrogenase complex (α-KDH) in the tricarboxylic acid cycle. Moreover, thiamine is involved in reduced glutathione generation and thus contributes to the counteraction of oxidative stress [31]. We identified about 189 cases reported worldwide [32,33,34,35,36,37,38,39,40,41,42,43,44,45,46,47], mostly characterized by a triad of non-type 1 diabetes mellitus, sensorineural deafness, megaloblastic anemia, optic nerve atrophy, retinal dystrophy, short stature and complete reversal of positions of major thoracic and abdominal organs (*situs inversus totalis*) [33]. We identified 24 patients in whom cardiac manifestation have been observed and whose cardiac symptom spectrum encompasses the following: DC, AF, barely visible P waves, RBBB, wandering pacemaker, endocardial cushion defect, BVH, VSD, (secundum) ASD, atrial dysrhythmias, DCM, congestive HF, supraventricular TC, TI (including that which is secondary to Ebstein’s anomaly), EA, subendocardial ischemia and ST-T changes consistent with diaphragmatic wall myocardial infarction [32,33,34,35,36,37,38,39,40,41,42,43,45,46,47] (Table 2, Supplementary Tables S2 and S3).

**Table 2:** Disorders of cytosolic transporters of sugars and sugar-linked metabolites presenting with cardiac manifestations (abbreviations described in Table 1).

Affected Gene	Affected Protein	Inheritance	Heart Defects & Manifestations			No. Patients Identified by Our Search	Ref.
			Cardiomyopathies	Structural Defects	Arrhythmogenic Disorders		
*SLC2A3*	Glucose transporter type 3 (GLUT3)	AR	-	ASD, PVSD	TC, HF	6	[22]
*SLC2A10*	Glucose transporter type 10 (GLUT10)	AR	CM, ICM, RDA	DAR, MVP, PPS	-	4	[28,29,30]
*SLC19A2*	THTR1 transporter	AR	BVH, DCM	DC, ECD, RBBB, VSD, ASD, EA, TI (Ebstein)	TC, AF, HF	24	[32,33,34,35,36,37,38,39,40,41,42,43,45,46,47]

### 3.2. Disorders of the Pentose Phosphate Pathway

The pentose phosphate pathway (PPP), also known as the pentose phosphate shunt, is a ubiquitous pathway highly conserved in living organisms, which branches from glucose 6-phosphate. It is essential for the synthesis of ribose-5-phosphate, a precursor to amino acid and nucleic acid production and NADPH, a metabolite critical to reduction–oxidation (redox) balance and detoxification of intracellular radical oxygen species [48,49]. The PPP is divided into two biochemical branches: an oxidative branch and a non-oxidative branch [12]. The oxidative branch converts glucose 6-phosphate into ribulose-5-phosphate with generation of CO_2_ and NADPH. The non-oxidative branch yields glycolytic intermediates, such as fructose 6-phosphate and glyceraldehyde 3-phosphate and sedoheptulose sugars, which contribute to the production of sugar phosphate precursors to amino acid synthesis and ribose-5-phosphate [12]. Under healthy growth conditions, the PPP regulates cell cycle progression, myelin formation and the maintenance of the structure of many organs. In pathologic conditions, the PPP is defective and cells may adopt alternative metabolic pathways to generate NADPH that do not depend on the immediate supply of glucose and are largely mediated by the activation of AMPK [50]. Of the three reported inborn errors of the PPP in our research, two were reported in association with cardiac manifestations (Table 3).

#### 3.2.1. TALDO Deficiency

Transaldolase deficiency (TALDOD) [OMIM:606003] is caused by AR-inherited homozygous or compound heterozygous mutations affecting the *TALDO1* gene on chromosome 11p15.5. Transaldolase is involved in the irreversible, non-oxidative part of the PPP, where pentose phosphates are recycled into hexose phosphates in concerted action with transketolase [51,52,53]. Therefore, TALDOD leads to the accumulation of polyols derived from PPP intermediates: arabitol, ribitol and erythriol. To date, only ten patients from three unrelated families have been reported [52]. Clinical manifestations include hydrops foetalis with oligoaminos, dysmorphia, severe recurrent anemia, hepatosplenomegaly and variable liver, renal and cardiac involvement [54]. Specifically, cardiac symptoms arise in early infancy and may either gradually improve or lead to infantile death or adult cirrhosis [54]. Fewer than 50 cases of TALDOD have been reported to date, of which we identified 35 patients described with congenital heart disease, VSD and/or ASD, BAV, DC, AC, CM, LVH and RVH, or TVR [53,55,56,57] (Table 3, Supplementary Tables S2 and S3).

**Table 3:** Disorders of the pentose phosphate pathway for which patients displaying cardiac manifestations have been reported (abbreviations described in Table 1).

Affected Gene	Affected Protein	Inheritance	Heart Defects & Manifestations			No. Patients Identified by Our Search	Ref.
			Cardiomyopathies	Structural Defects	Arrhythmogenic Disorders		
TALDO	Transaldolase (TALDO)	AR	CM, LVH	VSD, ASD, BAV, DC, MVP, TR	HF	35	[53,55,56,57]
G6PDH	Glucose-6-phosphate dehydrogenase (G6PDH)	XL	CD	PDA, PVSD, MVS, TR	HF	>300	[58,59,60,61,62,63,64,65,66,67] (selected) *

* For G6PDH deficiency our search produced over 300 entries (articles), thus only some meaningful articles were selected.

#### 3.2.2. G6PDH Deficiency

Glucose-6-phosphate dehydrogenase (G6PDH) deficiency [OMIM:300908] is one of the most common forms of enzyme deficiency and although prevalence estimations vary in different studies, it is considered to affect over 400 million people worldwide [58]. It is a dominant X-linked (XL) disorder caused by mutations in the *G6PDH* gene at location Xq28. G6PDH is the rate-limiting step of the oxidative PPP and catalyzes the conversion of glucose 6-phosphate to 6-phosphoglucolacetone, accompanied by NADPH production. In G6PDH deficiency, the first, irreversible step of the PPP is compromised, with consequential decreased production of NADPH in the hexose monophosphate pathway. Molecular investigations in mice revealed that G6PDH and NADPH levels are involved in modulating myocardial contractility under physiological and pathophysiological conditions by interacting with L-type Ca^2+^ channel activity [59]. The lack of the G6PDH enzyme leads to hemolysis and, when compensation is not possible, anemia develops. Patients are mostly asymptomatic however when exposed to triggers (drugs, infectious diseases and *fava* bean consumption), they may develop life threatening acute hemolytic anemia. Our literature search produced several reports of single or few G6PH-deficient patients describing with cardiac symptoms, which included: CD, HF, TVR, severe MVS, PVSD, PDA, coronary artery disease and ST-segment elevation [58,60,61,62,63,64,65,66] (Table 3). More recently, studies on large patient cohorts have also been performed to investigate whether patients with G6PDH-deficiency present a higher predisposition to cardiovascular diseases and the results are still controversial. For instance, in the study from Dore et al. [67], 324 elderly G6PDH-deficient patients (out of 1123) have been confirmed to have cardiovascular defects, while Meloni et al. [68] found that cardiovascular defects are less frequently present among G6PDH-deficient patients than in controls (11.8% vs. 18.6%, respectively). Moreover, several studies suggest that patients’ age plays a role in the development of cardiovascular symptoms in G6PDH deficiency [67,69]. This evidence has also been observed in G6PDX mice, which showed higher susceptibility to age-associated cardiac hypertrophy and ventricular dilation in response to myocardial infarction or pressure overload-induced heart failure [69,70]. Definitive clinical studies in large populations are needed to determine the effects of G6PDH deficiency on the development of cardiovascular disease and subsequent outcomes.

### 3.3. Disorders of Glycogen Metabolism

Glycogen storage diseases (GSDs) are a group of IMDs which arise from congenital defects that affect glycogen synthesis (glycogenesis) or breakdown (glycogenolysis) primarily in hepatic and muscle tissues [71]. Glycogen is a multibranched polysaccharide that acts as a readily mobilized storage form of glucose mostly in liver, muscle and heart cells to different extents [11]. When systemic glucose levels are low, for example during fasting, starvation or intense physical exercise, glycogen is mobilized and broken down to yield glucose molecules that are used by the muscle and cardiac tissues or released from the liver into the bloodstream to support the energy demand of several tissues, while it is directly used in the muscle to support contraction [71]. In a healthy situation, this process is highly controlled and serves as a buffer to maintain blood glucose levels. Conversely, when genetic defects lead to the inability of the body to store or break down glycogen, resulting in very low blood glucose levels during fasting periods, those defects are indicated as GSDs. GSDs are mostly AR-inherited and have been divided into subtypes based on genotypic and phenotypic heterogeneity. The lack of activity in glycogen processing enzymes result in a wide range of clinical manifestations including hypoglycemia, hypotonia, muscle weakness, myoglobinuria, hyperlipidemia, elevated liver aminotransferases, elevated creatine kinase, CMG and other cardiac manifestations [71]. Of the 14 GSDs described to date, eight have been found to present with cardiac manifestations (Table 4).

#### 3.3.1. GAA-GSD

Pompe disease is an AR GSD type IIa [OMIM:232300] with infantile onset, caused by acid maltase deficiency (AMD). AMD is due to a homozygous or compound heterozygous mutations in the *GAA* gene, localized on chromosome 17q25.3. Its prevalence is estimated at between 1/30,000–140,000 births worldwide, depending on the form [72]. *GAA* mutations result in the loss of function of the acid maltase enzyme and ultimately in intra-lysosomal accumulation [73]. Classically, GAA-GSD is classified in three forms. The infantile form (also known as “classic” Pompe disease) has neonatal onset and average life expectancy does not exceed one year of age. Clinically, it manifests with skeletal myopathy, muscle weakness, hepatomegaly and prominent CM and hypertrophic DCM that result in lethal cardiorespiratory failure [72,73,74]. The childhood/juvenile form (also known as “non-classical” infantile Pompe disease) has an onset at around six months of age and life expectancy longer than two years, as the clinical spectrum is similar to the infantile form but with milder cardiac involvement [72]. Lastly, the adult form has the mildest clinical presentation, mostly consisting of progressive proximal myopathy and usually without cardiac involvement [72].

Focusing on the non-classical form of Pompe disease, a comprehensive systematic review from van Kooten et al. [75] collected information on 750 Pompe patients and described several cardiac phenotypes reported significantly more frequently in these patients than in the normal population and these included DCM, HCM, sinus AT, AF, TC, VLD, prolonged QRS interval and QT interval, short PR interval, sick sinus syndrome, incomplete bundle branch block, LBBB, RBBB, LAE, AVB, LVH (in some cases with left ventricular outflow tract obstruction), VEFR, ventricular repolarization disorder, BVH, MVP and hypertension (Table 4).

Enzyme replacement therapy (ERT) is now a consolidated treatment for Pompe disease and can efficiently prevent deterioration of cardiac symptoms while partially helping to recover some functional dysfunction [73].

#### 3.3.2. GBE-GSD

Andersen’s disease [OMIM:232500], or AR GSD type IV, is caused by mutations in the *GBE1* gene at location 3p12, which encodes the glycogen branching enzyme (GBE, or 1,4-α-glucan branching enzyme type 1). This disorder alone accounts for 3% of all GSDs [76,77]. Loss of function of GBE translates to an accumulation of abnormal glycogen chains with fewer branch points, called polyglucosans. These polyglucosans aggregate in metabotoxic polyglucosan bodies that impair the function of organs and tissues, particularly the central neuromuscular system and liver. Clinical presentation of Andersen’s disease is vastly heterogenous. The most severe form presents as fetal perinatal neuromuscular disorder, which may include polyhydramnios causative of fetal akinesia deformation sequence that lead to arthrogryposis after birth [78]. After birth, patients develop severe muscular hypotonia and atrophy, along with cardiac symptoms often linked to deposition of amylopectin-like polysaccharides in the myocardium. Our systematic search produced 35 patients with GBE deficiency with cardiac involvement, including LVH, CMG, DCM or HCM, the latter two often reported as the cause of HF [76,77,79,80,81,82,83,84,85,86,87,88,89,90,91,92,93,94,95,96,97,98,99,100,101] (Table 4, Supplementary Tables S2 and S3). In most cases, patients do not survive past the neonatal period due to cardiorespiratory complications [102,103].

#### 3.3.3. GDE-GSD

Cori–Forbes disease [OMIM:232400] is an AR glycogen storage disorder (type III) caused by homozygous or compound heterozygous mutations affecting the amylo-α-1,6-glucosidase/4-α-glucanotransferase (*AGL*) gene located on chromosome 1p21. This gene encodes the glycogen debrancher enzyme (GDE), which is involved in glycogen degradation. This enzyme has two independent catalytic activities that occur at different sites on the protein: a 4-α-glucotransferase activity and an amylo-1,6-glucosidase activity. When genetic mutations cause a loss of function of GDE, accumulation of abnormal glycogen with short outer chains occurs, which often cause hypertrophy in the affected tissues. GSDIII has been traditionally divided into two subtypes: in GSDIIIa, the most common, the enzymatic deficiency affects the liver, heart and skeletal muscle, while in GSDIIIb, only the liver is affected [104]. Focusing on GSDIIIa, our systematic search produced 204 patients with cardiac involvement, predominantly represented by DCM and HCM, but also RVH, LVH, LVD, SH and heart murmur [80,105,106,107,108,109,110,111,112,113,114,115,116,117,118,119,120,121,122,123,124,125,126,127,128,129,130,131,132,133,134,135,136,137,138,139,140,141,142,143,144,145,146] (Table 4, Supplementary Tables S2 and S3).

#### 3.3.4. GYG1-GSD

GSD type XV [OMIM:613507] is a rare AR disorder caused by compound heterozygous mutations in the glycogenin type 1 (*GYG1*) gene on chromosome 3q24. The prevalence of GSD type XV is estimated to be at lower than 1/1,000,000. Glycogenin 1 is a glycosyltransferase that catalyzes the synthesis of short glucose polymers, indicated as (1,4-α-D-glucosyl)_n_ or polyglucosans, from UDP-glucose. These short polymers act as substrates for glycogen synthase and branching enzyme for the synthesis of new glycogen macro-polymers [102]. Clinical manifestations include profound glycogen depletion in skeletal muscle and abnormal accumulation in the heart. Seven patients have been reported with cardiac symptoms, including HCM, AT, VEFR, late-onset coronary artery disease and HF [147,148,149,150] (Table 4, Supplementary Tables S2 and S3). Endomyocardial biopsy often shows hypertrophic cardiomyocytes (suggestive of HCM) with enlarged nuclei, large centrally located vacuoles with periodic acid Schiff (PAS)-positive material and glycogen depletion in the cytoplasm [147,151].

#### 3.3.5. GYS1-GSD

GSD type 0b [OMIM:611556] is an AR disorder caused by homozygous mutations in the muscle glycogen synthase type 1 (*GYS1*) gene, located at 19q13.33 and has an estimated prevalence of <1/1,000,000 [152,153]. These mutations cause loss of function of the GYS1 protein, which leads to a lack of glycogen biosynthesis in skeletal muscle and the heart. This results in symptoms such as severe syncope (following even moderate exercise), hypoglycemia, muscle pain, lethargy, loss of consciousness, arrythmias and CM [153]. Furthermore, GYS1 deficiency compromises the cardiac ability to pump blood and increases the risk of cardiac arrest and sudden death during exercise [152,153,154]. Our search identified four patients affected by GYS1 deficiency [152,153]. In 2007, Kolberg et al. reported 3 siblings from consanguineous parents who showed severe cardiac symptoms with exercise intolerance [152] (Table 4, Supplementary Tables S2 and S3). In detail, the eldest sibling developed tonic–clonic seizures during infancy and died of cardiac arrest at age 10.5 years as result of thickened LV and HCM. The second sibling also exhibited signs of HCM, abnormal heart rate and pressure. Cardiac examination in the youngest sibling revealed LAE and DI at rest [152].

#### 3.3.6. LAMP2-GSD

GSD type II(b), more commonly known as Danon disease, [OMIM:300257] is a variant of Pompe disease with XLD inheritance, characterized by severe cardiac manifestations, along with skeletal and neurological symptoms, with estimated prevalence of <1/1,000,000. Genetically, it is caused by mutations of the *LAMP2* gene, localized on chromosome Xq24, which encodes a lysosome-associated membrane protein-2. LAMP2 is a critical component of lysosomal membranes and plays a role in autophagosome–lysosome fusion, lysosome biogenesis and lysosomal membrane permeabilization (LMP) [155,156]. Its loss of function results in a wide spectrum of symptoms, including vacuolar cardioskeletal myopathy. Classically, the onset in males is around 10 years old, but occurs later in females and to date it has been identified in over 20 families [157]. Two specific LAMP2 mutations have been associated with prominent hypertrophy and electrophysiological abnormalities [157,158]. Our systematic review resulted in 200 Danon patients predominantly showing severe HCM (less often DCM), CMG, Wolf–Parkinson–White syndrome with complete AVB and LBBB, syncope, AF and LVH (with ventricular preexcitation), which often led to death or heart transplant [110,157,158,159,160,161,162,163,164,165,166,167,168,169,170,171,172,173,174,175,176,177,178,179,180,181,182,183,184,185,186,187,188,189,190,191,192] (Table 4, Supplementary Tables S2 and S3).

#### 3.3.7. PRKAG2-GSD

Glycogen glycogen-associated cardiomyopathy [OMIM:602743] is an autosomal dominant GSD caused by mutations in the protein kinase, AMP-activated, noncatalytic, gamma-2 (*PRKAG2*) gene on chromosome 7q36.1, which is associated with Wolff–Parkinson–White syndrome (WPW) [OMIM:194200]. The affected protein is a serine/threonine AMP-activated protein kinase (AMPK) that in response to activation by several cellular stressors induces increased AMP production and ATP catabolism [193]. In 2001, two families with severe HCM were identified as PRKAG2 mutation carriers. Molecular studies in these patients suggested that, since AMPK provides a central sensing mechanism that protects cells from ATP exhaustion, disruption of energy homeostasis could be a unifying pathogenic mechanism in all forms of HCM [194]. Overall, we found 103 clinically affected patients with cardiac involvement (and skeletal muscle glycogenosis and myopathy in some cases), who were investigated often along with family members who were carriers of *PRKAG2* mutations [195,196,197,198,199,200,201,202,203,204,205]. Besides HCM due to defective glycogen storage, cardiac symptoms reported WPW (progressive dysfunction of the conduction system), conduction system degeneration (RBBB, LBBB, AVB), ventricular preexcitation, LVH, supraventricular TC, atrial premature beat, maximal left ventricular wall thickness stoke episodes and HF (Table 4, Supplementary Tables S2 and S3).

#### 3.3.8. RBCK1-GSD

This congenital disorder, also referred to as polyglucosan body myopathy-1 (PGBM1) [OMIM:610924], is an AR GSD caused by mutations on the RANBP-type and C3HC4-type zinc finger-containing 1 (*RBCK1*) gene located on chromosome 20p13 [206,207]. The encoded protein is an E3 ubiquitin ligase complex responsible for adding head-to-tail polyubiquitin chains to substrate proteins and it plays a role in NFkB and JNK signaling pathways [208]. Although the molecular mechanisms remain mostly elusive, deficiency of this enzyme induces accumulations of polyglucosan in cardiac and muscle tissues (also known as amylopectinosis). This peculiar characteristic has suggested a link between this disease and GSDs. Of 22 patients reported, 18 have been reported with severe cardiac manifestations. The first report from 2012 described three patients that, among several other symptoms, all developed CM with congestive HF in early childhood [207]. Following studies collectively reported 16 patients who developed progressive DCM, which led to heart transplants in nine cases and to death due to heart failure in two patients [206,209,210,211] (Table 4, Supplementary Tables S2 and S3).

#### 3.3.9. SLC37A4-GSD

Homozygous and heterozygous compound mutations of the *SLC37A4* gene on chromosome 11q23.3 cause an AR disorder that compromises both glycogen metabolism and glycosylation and thus can be defined both as glycogen storage disease type Ib (GSDIb) and as congenital disorder of glycosylation type IIw (CDG2W) [OMIM:619525]. This gene encodes glucose 6-phosphate translocase type I (G6PT1), which is responsible for regulating the rate-limiting step of glucose 6-phosphate transport through the membrane of the ER. Hence, G6PT1 functions as a glucose 6-phosphate receptor/sensor in ATP-mediated calcium sequestration in the ER lumen [212]. Biochemically, deficiency of G6PT1 leads to excessive fat and glycogen in the liver, kidneys and intestinal mucosa and lactic acidosis and profound abnormalities in the N-glycosylation of serum specific proteins [213]. Clinically, this disorder is characterized by liver dysfunction and hepatomegaly, renomegaly, neutropenia, hypoglycemia and coagulation defects [213,214]. Unlike GSD type II and III, the heart is not primarily affected in this GSDIb. The most common cardiovascular abnormality in patients is systemic hypertension, which usually occurs in the context of renal disease. We identified four patients reported with diagnosed SLC37A4 deficiency and cardiac abnormalities, manifesting as ToF, VSD, PPS and RVH [213,214] (Table 4, Supplementary Tables S2 and S3).

**Table 4:** Glycogen storage diseases for which patients displaying clinical cardiac manifestations have been reported (abbreviations described in Table 1).

Affected Gene	Affected Protein	Inheritance	Heart Defects & Manifestations			No. Patients Identified by Our Search	Ref.
			Cardiomyopathies	Structural Defects	Arrhythmogenic Disorders		
*GAA*	α-1,4-glucosidase (GAA)	AR	DCM, HCM, LVH, VEFR, BVH	VLD, LAE, LBBB, RBBB, AVB, MVP	AT, AF, TC, HF, LQTS	>300	[72,73,74,75] (selected) *
*GBE1*	Glycogen branching enzyme (GBE1)	AR	DCM, HCM, LVH	CMG	AT, HF	35	[76,77,79,80,81,82,83,84,85,86,87,88,89,90,91,92,93,94,95,96,97,98,99,100,101]
*AGL*	Glycogen debranching enzyme (GDE)	AR	DCM/HCM, LVH, RDH, LVD	SD	-	204	[80,105,106,107,108,109,110,111,112,113,114,115,116,117,118,119,120,121,122,123,124,125,126,127,128,129,130,131,132,133,134,135,136,137,138,139,140,141,142,143,144,145,146]
*GYG1*	Glycogenitype 1 (GYG1)	AR	HCM	-	VF, TC, HF	7	[147,148,149,150]
*GYS1*	Muscle glycogen synthase	AR	HCM, LVH	LAE	HF	4	[152,153]
*LAMP2*	Lysosome-associated membrane protein-2 (LAMP2)	XLD	DCM, HCM, CH	-	-	200	[110,157,158,159,160,161,162,163,164,165,166,167,168,169,170,171,172,173,174,175,176,177,178,179,180,181,182,183,184,185,186,187,188,189,190,191,192]
*PRKAG2*	AMP-activated protein kinase (AMPK)	AD	HCM, LVH, DI	AVB, LBBB, RBBB	HF, TC	103	[195,196,197,198,199,200,201,202,203,204,205]
*RBCK1*	E3 Ubiquitin ligase	AR	DCM	-	HF	16	[206,209,210,211]
*SCL37A4*	Glucose 6-phosphate translocase type I (G6PT1)	AD	RVH	ASD, VSD, ToF, PPS	HF	4	[213,214]

* For GAA deficiency our search produced over 300 entries (articles), thus only some meaningful articles were selected.

### 3.4. Congenital Disorders of Glycosylation

Glycosylation is a complex post-translational modification that consists in the attachment of one or more chains made of monosaccharides onto acceptor molecules, such as proteins and lipids. The attachment of these carbohydrate chains allows the acceptor molecules to acquire their functional three-dimensional folding and final physicochemical properties, such as solubility. Glycoconjugates, like glycoproteins, are critical for cell recognition and adhesion, cell migration, protease resistance and many other biological functions [14]. Glycomics-based studies of cardiac cells, also known as cardiomyocytes, have suggested that glycosylation is a critical process for the regulation and modulation of cardiac structural development and functionality [14].

Congenital disorders of glycosylation (CDGs) represent a clinically and genetically heterogenous group of rare monogenic disorders affecting the synthesis, processing, attachment and degradation of glycans. CDGs consist of more than 160 disorders [215], of which we identified 29 that clinically present with cardiac manifestations in patients (Table 5).

#### 3.4.1. Disorders Affecting N-Glycosylation

##### ALG3-CDG

Alpha-1,3-mannosyltransferase (ALG3) deficiency [OMIM:601110] is a CDG (type 1d) with AR inheritance and an estimated prevalence of <1/1,000,000 live births [216]. It is caused by a homozygous or compound heterozygous mutation in the *ALG3* gene on chromosome 3q27, which encodes the enzyme that is responsible for the addition of the 6th mannose to the dolichol-linked oligosaccharide in the endoplasmic reticulum [217]. About 44 patients have been reported to date [218], with a broad phenotypical spectrum that mainly includes neurological, skeletal, gastrointestinal and urogenital symptoms [217]. We identified 15 patients described with cardiac symptoms, which collectively involved HOCM, RDA, TVR, VSD/AVD, MVS, PDA, PFO, PDA, truncus arteriosus type II, poor biventricular function and congenital heart disease [216,218,219,220] (Table 5, Supplementary Tables S2 and S3).

##### ALG6-CDG

Alpha-1,3-glucosyltransferase deficiency [OMIM:603147] is a hyper-rare CDG (type Ic) caused by the loss of function of the enzyme encoded by the *ALG6* gene, located on chromosome 1p31. ALG6 enzyme, also called glucosyltransferase 1, catalyzes the addition of the first glucose residue to the growing lipid-linked oligosaccharide precursor of N-linked glycosylation. The CDG resulting from this deficiency manifests in muscular hypotonia, ataxia, motor developmental retardation and severe neurological involvement and more rarely retinal degeneration, deep vein thrombosis and pseudotumor cerebri [221]. However, one patient was reported with a novel mutation (c.482A>G; p.Y161C) and unusual presentation, including very mild neurological symptoms but the presence of DCM and LV dysfunction [221] (Table 5, Supplementary Tables S2 and S3).

##### ALG9-CDG

Alpha-1,2-mannosyltransferase (ALG9) deficiency [OMIM:607143] is a CDG (type Il) has a prevalence of <1/1,000,000 live births. It is caused by an AR loss-of-function mutation in the *ALG9* gene on chromosome 11q23. The encoded protein, alpha-1,2-mannosyltransferase (ALG9), catalyzes the transfer of the 7^th^ and 9^th^ mannose residues to the growing lipid-linked glycan chains in the endoplasmic reticulum [7,14]. The overall clinical spectrum of this CDG includes progressive microcephaly, hypotonia, developmental delay, hepatomegaly and drug-resistant infantile epilepsy. Additional features may include skeletal dysplasia and pericardial effusion. A total of 19 patients have been reported to date [222], of which 12 have been described as displaying cardiac symptoms, including TVR, BAV, SAI, ASD, pericardial effusion, RVD and, in one case, severely reduced biventricular function and PDA [14,219,222,223,224,225,226] (Table 5, Supplementary Tables S2 and S3).

##### ALG12-CDG

Alpha-1,6-mannosyltransferase (ALG12) deficiency [OMIM:607143] is another very rare AR (CDG type Ig) caused by a loss-of-function mutation in the *ALG12* gene on chromosome 22q13.33. The encoded protein is Dolichol-P-mannose: Man_7_GlcNAc_2_-PP-Dol-alpha-6-mannosyltransferase, responsible for the addition of the 8^th^ mannose residue on the immature glycan chain Man_7_GlcNAc_2_-PP-Dol. This CDG is characterized by generalized hypotonia, feeding difficulties and facial dysmorphism, which can present along with skeletal anomalies, seizures, and cardiac anomalies in some cases [14,227,228,229]. To date, fewer than 15 cases have been reported in the literature, of which nine displayed HCM, PVSD, misalignment of the interventricular septum, deviation of the left ventricular outflow tract, PDA, VSD and AT [14,227,228,230] (Table 5, Supplementary Tables S2 and S3). One of these patients died before the age of two as a result of cardiorespiratory failure associated with CM [227].

##### GMPPB-CDG

GDP-mannose pyrophosphorylase B (GMPPB) deficiency [OMIM:615320] is an AR-inherited CDG caused by homozygous or compound heterozygous mutations in the *GMPPB* gene on chromosome 3p21.31. This gene encodes the beta subunit of the enzyme that catalyzes the conversion of mannose-1-phosphate and GTP to inorganic diphosphate and GDP-mannose (GDP-Man), which is an essential mannosyl-donor required for N-, O- and C-linked glycosylation [231]. Pathological reduction of GDP-Man manifests similarly to other dystroglycanopathies, with muscle phenotypes ranging from severe congenital muscular dystrophy to Limb–Girdle Muscular Dystrophy (LGMD) [232]. To date, fewer than 15 patients have been described with GMPPB deficiency and mostly displayed severe muscle phenotypes, hypotonia, microcephaly, epilepsy, strabismus, nystagmus and cataracts. Our search identified four patients reported with cardiac clinical features, specifically LVD, RBBB, sino-atrial block with atrial ectopics, aberrant ventricular conduction and cardiorespiratory compromise [232,233] (Table 5, Supplementary Tables S2 and S3). In addition, one patient was reported by Oestergaard et al. with VEFR; however, no more details on underlying cardiac causes were provided [234].

##### NPL-CDG

N-acetylneuraminate pyruvate lyase (NPL) deficiency [OMIM:611412] is a very rare CDG with AR inheritance due to compound heterozygous mutations resulting in the loss of function of the *NPL* gene. NPL enzyme, also known as sialic acid aldolase, regulates the cellular concentration of sialic acid by catalyzing SA conversion into N-acetylmannosamines (ManNAc) and pyruvate. Sialic acids are essential components of glycoproteins and glycolipids, which enable critical cellular processes. Sialic acid catabolism has been proven recently to be important for cardiac and skeletal muscle function and development [235]. In fact, NPL-CDG leads to NPL myopathy, exercise intolerance and elevated urinary sialic acid [235]. ManNAc and N-acetylmannosamine 6-phosphate (ManNAc-6P) were found to be significantly reduced in the cells of two siblings with compound heterozygous mutations in the *NPL* gene, pointing to affected NPL enzyme activity and defected SA catabolism [236]. Cardiac symptoms were observed in one of the patients, who developed fetal AT with hydrops and after birth was diagnosed with progressive DCM, LVH, VEFR and cardiac arrest [236] (Table 5, Supplementary Tables S2 and S3). Interestingly, in an NPL-knockdown zebrafish model, the cardiac phenotype was rescued with ManNAc, suggesting the possibility of monosaccharide replacement therapy in human patients [236].

##### PGM1-CDG

Genetic mutations affecting the phosphoglucomutase 1 (*PGM1*) gene located on chromosome 1p31.3 are causative of CDG type It [OMIM:614921], a rare disorder with AR inheritance and unknown prevalence in the population. PGM1 protein is an enzyme belonging to the phosphohexose mutase family. Although several PGM isoenzymes have been described in humans, PGM1 alone accounts for 90% of phosphoglucomutase activity in the body [237]. Specifically, PGM1 catalyzes the transfer of phosphate between position 1 and 6 of glucose. Glucose 6-phosphate flows mostly into glycolysis for energy production, while glucose 1-phosphate is the substrate for UDP-glucose synthesis, which is the building block of glycogen (PGM1-CDG is also considered a glycogen storage disorder) and of glycosylation [238]. PGM1-CDG is characterized by high clinical heterogeneity and multi-organ involvement, but the most frequently diagnosed symptoms are cleft palate/uvula, hepatopathy, growth delay, endocrine deficiency, exercise intolerance, myopathy (with or without rhabdomyolysis) and cardiac defects (which proved fatal in some cases) [239]. To date, about 60 patients with confirmed PGM1 deficiency have been reported [239,240,241,242,243]. Of these, 30 have been reported with cardiac involvement and at least six required heart transplantation [239,241,243]. Cardiac defects vary in type and severity and include: DCM (in some cases RCM), LVD, LVH, VSD, AC, MP, AT, AF, CMG, heart rhythm alterations and, in the most severe case, cardiac arrest [14,239,241,243,244,245,246,247,248,249,250,251,252,253,254,255,256,257] (Table 5, Supplementary Tables S2 and S3). A potential molecular link between PGM1 and cardiac symptoms (particularly DCM) was proposed by Arimura et al. [258], who found that in stressed rat myocardium, PGM1 binds to an anchoring protein named Z-band alternatively spliced PDS-motif protein (ZASP, homolog of LDB3 in human) and suggested this to be a compensatory cardioprotective mechanism which would be compromised in PGM1-deficient patients.

##### PMM2-CDG

Phosphomannomutase 2 (PMM2) deficiency [OMIM:212065] is the most frequently diagnosed CDG. It has AR inheritance and its estimated incidence reaches up to 1/20,000 newborns and it affects over 800 patients worldwide [215,240]. It is caused by homozygous or heterozygous compound mutations in the *PMM2* gene on chromosome 16p13. The affected PMM2 enzyme converts mannose-6-phosphate to mannose-1-phosphate, which is the immediate precursor of GDP-Mannose. PMM2-CDG can be classified into three forms: the infantile multisystemic form, the late infantile/childhood form presenting with ataxia and intellectual disability and the adult form that presents with stable disability. Clinically, *PMM2* mutations cause psychomotor delay, seizures, cerebellar hypoplasia, coagulopathy and, in the most severe (yet rare) cases CM and other cardiac symptoms. Through our research, we found 70 PMM2-CDG patients described with HCM (more rarely DCM), ToF, ASD, PDA, PFO, pericarditis and pericardial effusion and truncus arteriosus, but also PPS, vascular ring anomaly based on a right aortic arch and aberrant left subclavian artery have also been reported [14,230,243,259,260,261,262,263,264,265,266,267,268,269,270,271,272,273,274,275,276,277,278,279,280,281,282,283,284] (Table 5, Supplementary Tables S2 and S3).

#### 3.4.2. Disorders Affecting O-Glycosylation

##### B3GALTL-CDG

Enzymatic deficiency of O-Fucose-specific β-1,3-N-glucosyltransferase (*B3GALTL*, sometimes referred to as *B3GLCT* or *B3GTL*) [OMIM:261540], also known as Peters plus syndrome, is a rare AR disorder that presents with syndromic developmental defects mostly affecting the eye. It is caused by mutations in the *B3GALTL* gene that encodes an O-Fucose-specific β-1,3-N-glucosyltransferase responsible for transferring glucose to fucose with a β-1,3 linkage, thus contributing to the elongation of O-linked fucosylglycans on thrombospondin type-1 repeats (TSRs) of several proteins [285]. It is characterized by a variable phenotype including Peters anomaly (corneal defects), short limbs, characteristic facial features, mild to severe mental delay and genitourinary system disorders [14]. Our search resulted in the identification of 19 patients displaying cardiac anomalies, predominantly ASD, VSD, HoLV and, in a few cases, cardiac murmur, absence of right pulmonary vein and BPV [14,286,287,288,289,290,291,292] (Table 5, Supplementary Tables S2 and S3).

##### B3GAT3-CDG

Beta-1,3-glucuronyltransferase 3 (B3GAT3) deficiency [OMIM:245600], also known as Larsen-like syndrome, is a rare AR and idiopathic CDG with a mutation in the *B3GAT3* gene on chromosome 11q12.3 [14]. The affected beta-1,3-glucoronyltransferase 3 protein is a member of the glucoronyltransferase family, which exhibits strict acceptor specificity, recognizing nonreducing terminal sugars and their anomeric linkages—a critical step for proteoglycan synthesis. B3GAT3-CDG is characterized by skeletal dysplasia, multiple joint dislocations, joint laxity and other alterations of connective tissue, short stature, craniofacial dysmorphism, limb malformations, ocular defects, and cardiac symptoms [293]. Overall, we found 32 patients reported with this CDG, of which 15 exhibited variable cardiac presentations, including BAV, DAR, VSD, ASD, MVP, PDA and PPS [14,293,294,295,296,297,298,299,300,301] (Table 5, Supplementary Tables S2 and S3).

##### FKRP-CDG

Muscular dystrophy–dystroglycanopathy type C5 [OMIM:607155] (also called limb–girdle muscular dystrophy type 2I, or more recently type R9), muscular dystrophy–dystroglycanopathy type A5 (also known as Walker–Warburg syndrome) [OMIM:613153] and muscular dystrophy–dystroglycanopathy type B5 [OMIM:606612] are AR dystroglycanopathies caused by mutations in the fukutin-related protein gene (*FKRP*) on chromosome 19q13.32. The encoded protein, FKRP, is a ribitol-5-phosphate transferase involved in the functional glycosylation of α-dystroglycan (α-DG), which is a significant component in the link between the cytoskeleton and the extracellular matrix [302]. Mutations causing loss of function in FKRP lead to a broad spectrum of dystroglycanopathy-associated symptoms, primarily affecting the development of the muscles and heart, but also the brain and eyes. FKRP-linked disorders have been defined as the most severe forms of dystroglycanopathy and it has been suggested that a full loss of function of this protein results in embryonic lethality [303]. Our systematic search identified 220 FKRP-deficient patients with cardiac involvement [14,304,305,306,307,308,309,310,311,312,313,314,315,316,317,318,319,320,321,322,323,324,325,326,327,328,329] (Supplementary Tables S2 and S3). Cardiac manifestations are common clinical symptoms of FKRP-CDG, are especially severe in patients with homozygous mutations and can cause dyspnea, peripheral edema and cardiac arrest. These symptoms include DCM, LVD, VEFR (also linked to reduced cardiac torsion), ventricular extrasystoles, RBBB leading to HF; less frequently, LBBB, TGA and valvular defects have also been reported (Table 5).

##### FKTN-CDG

Fukuyama congenital muscular dystrophy [OMIM:253800] is a rare AR CDG originating from mutations in the fukutin (*FKTN*) gene located on chromosome 9q31.2. The estimated prevalence of this disorder has been estimated in Japan at between 6–11/100,000 live births, while estimations for other countries are still not available. Fukutin is responsible for the addition of ribitol-5-phosphate to a special type of glycan named called α-dystroglycan, which is usually conjugated to membrane proteins and lipids. α-dystroglycans are essential for the maintenance of muscle integrity, cortical histogenesis and normal ocular development. Therefore, defects in the FKTN gene lead to a congenital progressive muscular dystrophy characterized by brain malformation, dystrophic changes in skeletal muscle, severe intellectual deficit, epilepsy, and motor impairment [14,330]. Other features with later onset include myopathic facial appearance, pseudohypertrophy of the calves and forearms and progressive cardiac involvement [330]. Cardiac symptoms have been more often reported during the second decade of life, especially in patients with subtypes caused by p.Q358P and p.R179T mutations, who experience DCM with minimal muscle weakness [14]. Our systematic search results in 77 patients with FKTN deficiency and cardiac manifestations, encompassing DCM, VD, DI, SI, AF, PFO, double subaortic ventricular defect, HoLV, PPS, MF and infundibular TGA (with no innominate vein) [14,185,330,331,332,333,334,335,336,337,338,339,340,341,342,343] (Table 5, Supplementary Tables S2 and S3).

##### POMT1-CDG

Muscular dystrophy–dystroglycanopathy type A1 [OMIM:236670] is another severe AR congenital muscular dystrophy with a prevalence of <1/1,000,000. The mutation is found in the *POMT1* gene on chromosome 9q34.13. The protein encoded by this gene is O-mannosyltransferase 1 which catalyzes the first step in O-mannosyl glycan synthesis with attaching mannose to the serine or threonine residue of α-dystroglycans (α-DG) via O-glycosyl linkage [344]. O-mannosyltransferase 1 is localized in the ER and the structural role of α-DGs is in muscle fiber integrity, connecting the dystrophin–glycoprotein complex to the ECM. Defects in this enzyme result in reduced α-DG glycosylation in skeletal muscles, which leads to Walker–Warburg syndrome (WWS), congenital muscular dystrophy (CMD) and LGMD type 2 [345]. Symptoms usually include brain and eye malformations, severe mental disability, early death and varying degrees of severity [14]. In total, five patients have been described in the clinical literature as also displaying cardiac features. In the cohort of Pane et al. [321], only one POMT1-deficient patient was described with DCM, similar to the patient from Devisme et al. [346]. Instead, Bello et al. [345] reported four POMT1-deficient patients with wider cardiac manifestation: LVD, VEFR and LVH with moderate-to-severe SI (Table 5, Supplementary Tables S2 and S3).

##### POMT2-CDG

Muscular dystrophy–dystroglycanopathy type A2 [OMIM:613150] is a rare AR dystrophy that occurs as result of mutations in the O-mannosyltransferase 2 (*POMT2*) gene on chromosome 14q24.3. This gene encodes a protein called O-mannosyltransferase 2, which requires the activity of its homolog, POMT1, to initiate O-mannosylglycan synthesis in the endoplasmic reticulum, making it essential for the glycosylation of α-DGs [347]. Clinically, POMT2 deficiency is often associated with a wide range of clinical involvement, ranging from severe muscle–eye–brain disease and Walker–Warburg syndrome to limb–girdle muscular dystrophy without structural brain or ocular involvement. Although cardiovascular anomalies are thought to be uncommon in congenital muscular dystrophy, we identified seven patients reported with aortopathy (ascending aorta, AD and DAR, dilation of the annulus and sinotubular junction), reduced LV systolic function, LVH (non-progressive) and DCM [14,321,346,348,349] (Table 5, Supplementary Tables S2 and S3).

##### XYLT2-CDG

Spondyloocular syndrome (SOS) [OMIM:605822] is a rare AR CDG caused by mutations in the *XYLT2* gene located on chromosome 17q21.33. The affected protein, xylosyltransferase 2 (XYLT2), catalyzes the first step in the biosynthesis of chondroitin sulfate, heparan sulfate and dermatan sulfate proteoglycans. Proteoglycans are present in almost all ECMs of connective tissues and derive their major biochemical function from the physiochemical characteristics of the glycosaminoglycan component of the molecule, which provide hydration and swelling pressure to the tissue to withstand compressional forces [350,351]. Characteristic symptoms of SOS are bone fractures, cataracts, hearing loss, retinal detachment, and neurological defects. To date, only 22 patients have been reported [352]. Of these, three patients from two unrelated families were diagnosed with bone fragility, learning disabilities and cardiac symptoms, including ASD, MVP, AVD and mild MI [14,353,354] (Table 5, Supplementary Tables S2 and S3).

#### 3.4.3. Dolichol-Phosphate Synthesis Defects

##### DOLK-CDG

Dolichol kinase 1 (DOLK, or DK1) deficiency [OMIM:610768] is an AR CDG caused by homozygous or heterozygous compound mutations in the *DOLK* gene on chromosome 9q34.11. Its prevalence is estimated as <1/1,000,000 live births. DOLK is one of the enzymes involved in the de novo biosynthesis of dolichol phosphate mannose (Dol-P-Man). Dol-P-Man is an essential glycosyl carrier lipid for C- and O-mannosylation and N- and O-linked glycosylation of proteins and for the biosynthesis of glycosyl phosphatidylinositol anchors in the ER. Loss of function of this kinase clinically manifests with muscular hypotonia, ichthyosis, nervous system symptoms, and cardiac defects, predominantly CMs [355]. In most cases, patients required heart transplant or died due to heart complications. In total, 26 patients with DOLK-CDG with different cardiac manifestations were described in the literature: DCM and more rarely HCM, severe BVD, mild/severe CD, LVD, HF (acute congestive), non-sustained ventricular TC, BR, AT, CMG with right deviation of the heart, PDA, VSD, myocyte hypertrophy and interstitial fibrosis [14,227,355,356,357,358,359,360] (Table 5, Supplementary Tables S2 and S3).

##### DPM3-CDG

Limb–girdle muscular dystrophy–dystroglycanopathy type C15 (MDDGC15) [OMIM:612937] is an AR CDG caused by mutation on the dolichol-phosphate mannose synthase subunits 3 (*DPM3*) gene on chromosome 1q22. DPM3, together with DPM1 and DPM2, forms the DPM complex, which is responsible for the production of mannosyl donors for glycosylphosphatidylinositols, N-glycan synthesis, and protein O-/C-mannosylation [361]. Loss of function due to *DPM3* gene mutations clinically results in a rare type of limb–girdle muscular dystrophy–dystroglycanopathy, presenting with progressive proximal muscle weakness and DCM. Out of the 11 patients reported to date, four were described with DCM as the most predominant cardiac symptom and less frequently with mild LVD and LVRWMA [361,362,363] (Table 5, Supplementary Tables S2 and S3).

##### MPDU1-CDG

Mutations in the mannose-phosphate-dolichol utilization defect 1 (*MPDU1*) gene lead to a very rare AR CDG (type If). This gene, located on chromosome 17p13, encodes an ER membrane protein essential for the flipping of DPM and dolichol-phosphate-glucose (DPG) across the ER membrane and for the regulation of DPM and DPG within the ER lumen [364]. When defective, the synthesis of glycosylphosphatidylinositols and of lipid-linked oligosaccharides (LLO) is compromised, the latter resulting in the lack of complete N-glycans. MPDU1-CDG patients are mostly characterized by epilepsy, psychomotor retardation and skin abnormalities. Furthermore, four MDPU1-CDG patients out of six found in the literature shown either DCM or NCM [364,365,366] (Table 5, Supplementary Tables S2 and S3). In addition, the infantile patient described by Thiel et al. [365] had an older brother, who suffered from an undefined neonatal-onset disease with facial dysmorphism, skin ichthyosis and cardiac malformations (including *truncus arteriosus communis*) that died during neonatal cardiac surgery.

##### SRD5A3-CDG

Dolichol Steroid 5 α-reductase 3 (SRD5A3) deficiency [OMIM:612379] is a rare X-linked CDG resulting from mutations in the *SRD5A3* gene on chromosome 4q12. Loss of function of SRD5A3 enzyme compromises the conversion of polyprenol into dolichol, a necessary substrate for the beginning of multiple glycosylation processes [367]. Phenotypes in SRD5A3-CDG are highly variable and include ocular anomalies, mental retardation, cerebellar malformations and coagulation defects. Although cardiac involvement is sporadic, our search identified seven patients exhibiting heart symptoms, specifically CM, palpitations, AT, (secundum) ASD, LQTS, TGA and PFO [14,368,369,370,371] (Table 5, Supplementary Tables S2 and S3).

#### 3.4.4. Glycosylphosphatidylinositol (GPI)-Anchor Biosynthesis Defects

##### PIGA-CDG

Multiple congenital anomalies–hypotonia–seizures syndrome-2 (MCAHS2) [OMIM: 300868], caused by mutations on the phosphatidylinositol-glycan-anchor biosynthesis class A (*PIGA*) gene, is an idiopathic X-linked recessive (XL) neurodevelopmental disorder. Mutations in the *PIGA* gene on chromosome Xp22.2 2 lead to the loss of function of the PIGA protein, which participates in the synthesis of N-acetylglucosaminyl phosphatidylinositol on the ER membrane. This reaction represents the first step in the GPI anchor synthesis, and when deficient, it results in defective glycan synthesis [372]. PIGA-CDG has been identified in over 100 patients and is mainly characterized by dysmorphic features, neonatal hypotonia, early-onset myoclonic seizures and variable congenital anomalies affecting the urinary system and central nervous system [14,372,373,374]. Via our search, we identified 19 patients reported with a wide range of cardiac clinical features, namely ASD, CM, AR, PFO, atrial septal aneurysm, BAV, mildly AD (ascending), and first-degree AVB; in some cases these symptoms became the primary cause of spontaneous death due to cardiac arrest, especially in childhood [372,373,374,375,376,377] (Table 5, Supplementary Tables S2 and S3).

##### PIGL-CDG

Coloboma–congenital heart disease–ichthyosiform dermatitis–mental retardation–ear anomalies (CHIME) syndrome [OMIM:280000] is an AR CDG resulting from mutations in the phosphatidylinositol glycan anchor biosynthesis class L (*PIGL*) gene on chromosome 17p11.2. The loss of function of PIGL enzyme disrupts the second step of GPI synthesis in the ER, namely the de-N-acetylation of the N-acetylglucosaminylphosphatidylinositol (GlcNAc-PI), which results in the disruption of glycan synthesis and unoccupied glycosylation sites on proteins and other molecules. PIGL-CDG presents as a multisystemic disorder clinically characterized by colobomas, migratory ichthyosiform dermatosis, mental retardation, ear anomalies and congenital heart defects. Concerning the latter, to date eight patients have been reported with cardiac manifestations, ranging from TGA, HSS and ToF to VSD with pulmonary hypertension, DOV, PPS and systolic murmur [14,378,379,380,381] (Table 5, Supplementary Tables S2 and S3).

##### PIGN-CDG

Multiple congenital anomalies–hypotonia–seizures syndrome 1 (MCAHS1) [OMIM:614080] is an AR CDG due to mutations in the phosphatidylinositol glycan anchor biosynthesis class N (*PIGN*) gene. This gene encodes the GPI ethanolamine phosphate tranferase-1, which is also involved in GPI-anchor synthesis on the ER membrane. The backbone of GPI synthesis is assembled by the coordinated addition of sugar and phosphoethanolamine (EtNP) components to phosphatidylinositol. The disruption of this reaction leads to neonatal hypotonia, seizures, multiple congenital anomalies, and often premature death [14]. A total of 18 patients have been reported with heart defects in the form of VSD/ASD, PVSD, DC, LV noncompaction (NCM), PDA, ToF, PPS, PFO, RVD, DAR, OA and reduced left ventricular inotropy [14,382,383,384,385,386,387,388] (Table 5, Supplementary Tables S2 and S3).

##### PIGT-CDG

Multiple congenital anomalies–hypotonia–seizures syndrome 3 (MCAHS3) [OMIM:615398] is an AR CDG that originates from homozygous mutations on chromosome 20q13.12 in the phosphatidylinositol-glycan biosynthesis class T (*PIGT)* gene. The encoded enzyme is a subunit of a heteropentameric transamidase complex involved in the attachment of proteins to the GPI anchor, which functions as a plasma membrane anchor for extracellular proteins [389,390]. Fewer than 30 patients have been reported as having this disease, which is mostly characterized as manifesting with hypotonia, delayed psychomotor development, seizures, dysmorphic facial features, decreased serum alkaline phosphatase, kidney defects and skeletal abnormalities. Kvarnung et al. reported three patients belonging to a consanguineous family with PIGT-CDG manifesting the cardiac symptoms RCM, minor PDA and cardiac disease [389]. Later reports described five other patients with heart involvement, which further widened the cardiac symptom spectrum to other heart defects such as PFO and an atrial septal aneurysm [391,392] (Table 5, Supplementary Tables S2 and S3).

##### PIGV-CDG & PIGO-CDG

Homozygous and compound heterozygous mutations affecting the genes encoding phosphatidylinositol glycan (GPI) anchor biosynthetic enzymes class V (*PIGV*, located on chromosome 1p36.11) and class O (*PIGO*, located on 9p13.3) cause two very rare CDGs that manifest with facial dysmorphism, skin abnormalities, mental retardation, epilepsy and gastrointestinal defects. PIGV is localized in the ER, where it transfers the second mannose to the GPI backbone [393]. PIGO, together with another enzyme called PIGF, instead catalyzes the attachment of an ethanolamine phosphate to the third mannose of the three-mannosyl glycan core [394].

From our search, we identified one PIGV-deficient patient and three PIGO-deficient patients reported with cardiac symptoms. The PIGV-deficient patient and one of the PIGO-deficient patients were described with ASD, whilst the second and third PIGO-deficient patients exhibited ToF and TC, respectively [393,395,396,397] (Table 5, Supplementary Tables S2 and S3).

#### 3.4.5. COG Complex Defects

##### COG1-CDG & COG7-CDG

Genetic defects of the component of oligomeric Golgi complex 1 (COG1) [OMIM:611209] and complex 7 (COG-7) [OMIM:608779] genes lead to AR CDG type IIg and CDG type IIe, respectively. The *GOG1* gene is located on chromosome 17.q25.1, while the *COG7* gene is on chromosome 16p12.2. Glycoprotein modification and intracellular transport are key functions of the Golgi apparatus (GA), which depend on multiprotein complexes such as the Golgi transport complex, LDLC complex and SEC34 complex. These complexes participate in glycosylation reactions and vesicular transport. Together they are termed the conserved oligomeric Golgi complex (COG). COG1 and COG7 are thus necessary for GA structure and activity. To date, fewer than ten COG1-CDG patients have been described, presenting with dwarfism, facial dysmorphism, microcephaly and psychomotor delay. Of these, we identified four cases with HCM, PFO, SD, PMV and pulmonary hypertension [14,398,399,400,401] (Table 5, Supplementary Tables S2 and S3). For COG7-CDG eight patients were found in total, characterized by dysmorphic features and liver, gastrointestinal, skeletal and neurologic involvement. Cardiac involvement was found in six cases and included PVSD, (secundum) ASD and TI [14,402,403,404] (Table 5, Supplementary Tables S2 and S3).

#### 3.4.6. V-ATPase Complex Defects

##### ATP6V1A-CDG & ATP6V1E1-CDG

Defects of ATPase H^+^-transporting V1 subunit A (*ATP6V1A*) gene on chromosome 3q13.31 are causative of an AR CDG also known as cutis laxa type IId [OMIM:617403]. The acidification of endosomes, lysosomes, GA and other intracellular organelles are dependent on the vacuolar-type H^+^-ATPase (V-ATPase), a multimeric complex acting as an ATP-dependent protein pump, of which the protein encoded by ATP6V1A is a subunit. This complex is critical for the transport of hydrogen ions across the plasma membrane into the extracellular space and protein glycosylation. An imbalance in this process leads to acidification. So far, 4 patients have been reported, of which two exhibited SD (with tortuous aortic arch), LQTS with incomplete RBBB, HCM and progressive cardiac failure [14,405,406] (Table 5, Supplementary Tables S2 and S3). Likewise, mutations affecting the *ATP6V1E1* gene on chromosome 22q11.21 encoding another subunit of this complex are the cause of an AR inherited CDG, known as cutis laxa type IIC [OMIM:617402]. This CDG presents more severe cardiac manifestations. Out of six reported patients, five were described with a variety of cardiac symptoms: severe DAR, PFO, SD, MVR, TVR, HCM, AI and TI, RHHS with HoRV, TVS, small PDA, MVP, RVD with reduced diastolic compliance (DI) and RBBB [14,405,407] (Table 5, Supplementary Tables S2 and S3).

**Table 5:** Congenital disorders of glycosylation for which patients displaying cardiac manifestations have been reported (abbreviations described in Table 1).

Affected Gene	Affected Protein	Inheritance	Heart Defects & Manifestations			No. Patients Identified by Our Search	Ref.
			Cardiomyopathies	Structural Defects	Arrhythmogenic Disorders		
*Defects of N-Glycosilation*							
*ALG3*	Dolichol-P-mannose: Man5GlcNAc2-PP-dolichol mannosyltransferase	AR	HOCM, RDA, TR	VSD, AVD, PFO, PDA, MVS	-	15	[200,201,202,203,204,205,206,207,208,209,210,211,212,213,214,215,216,216,217,218]
*ALG6*	α-1,3-glucosyltransferase	AR	DCM, LVD	-	-	1	221
*ALG9*	α-1,2-mannosyltransferase	AR	RVD	ASD, BAV, SAI, PDA, TVR	-	12	[14,219,222,223,224,225,226]
*ALG12*	Dolichol-P-mannose: Man7GlcNAc2-PP-dolichol mannosyltransferase	AR	HCM, PVSD	PDA, SD, VSD, PFO	AT, HF	9	[14,227,228,230]
*GMPPB*	GDP-mannose pyrophosphorylase B (GMPPB)	AR	VD, LVD, VEFR	RBBB	-	5	[232,233,234]
*NPL*	N-acetylneuraminate pyruvate lyase (NPL)	AR	DCM, LVH, VEFR	-	AT, HF	1	236
*PGM1*	Phosphoglucomutase 1 (PGM1)	AR	DCM, RCM, LVD, LVH, AC, CMG	VSD, MP	AT, AF, HF	30	[14,239,241,243,244,245,246,247,248,249,250,251,252,253,254,255,256,257]
*PMM2*	Phosphomannomutase 2 (PMM2)	AR	HCM, ToF	ASD, PDA, PFO, PPS	-	70	[14,230,243,259,260,261,262,263,264,265,266,267,268,269,270,271,272,273,274,275,276,277,278,279,280,281,282,283,284]
*Defects of O-Glycosilation*							
*B3GALTL*	O-fucose-specific β-1,3-N-glucosyltransferasen (B3GALTL)	AR	HoLV	ASD, VSD, BVP	-	19	[14,286,287,288,289,290,291,292]
*B3GAT3*	β-1,3-glucuronyltransferase 3 (B3GAT3)	AR		BAV, VSD, ASD, MVP, PDA, PPS, DAR	-	15	[14,293,294,295,296,297,298,299,300,301]
*FKRP*	Fukutin-related protein (FKRP)	AR	LVD, LVRWMA, VEFR, DCM	VSD, TI, RBBB, TGA	HF	220	[14,304,305,306,307,308,309,310,311,312,313,314,315,316,317,318,319,320,321,322,323,324,325,326,327,328,329]
*FKTN*	Fukutin (FKTN)	AR	DCM, VEFR, CD	PPS, TGA, CMG	-	77	[14,183,330,331,332,333,334,335,336,337,338,339,340,341,342,343]
*POMT1*	O-mannosyltransferase 1 (POMT1)	AR	DCM, VD, VEFR, LVH, SI	-	-	5	[14,321,345,346]
*POMT2*	O-mannosyltransferase 2 (POMT2)	AR	LVH, DCM, VEFR	AD, DAR	-	7	[14,321,346,348,349]
*XYLT2*	Xylosyltransferase 2 (XYLT2)	AR	-	ASD, AVD, MVP	-	3	[14,353,354]
*Dolichol-phosphate synthesis defects*							
*DOLK*	Dolichol kinase (DOLK)	AR	DCM, HCM, BVD, CD, LVD	CMG, PDA, VSD	HF, TC, BR, AT	26	[14,227,355,356,357,358,359,360]
*DPM3*	Dolichol-phosphate mannose synthase subunit 3 (DPM3)	AR	DCM, LVD, LVRWMA	-	-	3	[361,362,363]
*MPDU1*	Mannose-phosphate-dolichol utilization defect 1 (MPDU1)	AR	DCM, NCM	-	-	4	[364,365,366]
*SRD5A3*	Steroid 5 α-reductase 3 (SRD5A3)	AR	CM	ASD, TGA, PFO	AT, LQTS	7	[14,368,369,370,371]
*Glycosylphosphatidylinositol anchor olichol-phosphate synthesis defects*							
*PIGA*	Phosphatidylinositol glycan anchor biosynthesis class A protein (PIGA)	XL	HCM	AD, BAV, PFO, ASD	AR. HF	19	[14,372,373,374,375,376,377]
*PIGL*	Phosphatidylinositol glycan anchor biosynthesis class L protein (PIGL)	AR	-	HSS, TGA, ToF, VSD, DOV, PPS	-	8	[14,378,379,380,381]
*PIGN*	Phosphatidylinositol glycan anchor biosynthesis class N protein (PIGN)	AR	NCM, RVD	DAR, PVSD, DC, PDA, PFO, ToF, PPS, DC, VSD, ASD, OA	-	18	[14,383,384,385,386,387,388]
*PIGT*	Phosphatidylinositol glycan anchor biosynthesis class T protein (PIGT)	AR	RCM	PDA, PFO, VSD, ASD	-	8	[14,389,391,392]
*PIGV*	Phosphatidylinositol glycan anchor biosynthetic enzymes class V (PIGV)	AR	-	ASD	-	1	[393]
*PIGO*	Phosphatidylinositol glycan anchor biosynthetic enzymes class O (PIGO)	AR	-	ASD, ToF	TC	2	[395,396,397]
*COG complex defects*							
*COG1*	Subunit 1 of the COG complex in Golgi trafficking (COG1)	AR	HCM	PFO, ASH, AI, PMV	-	4	[14,398,399,400,401]
*COG7*	Subunit 7 of the COG complex in Golgi trafficking (COG7)	AR	-	PVSD, TI, ASD	-	6	[14,398,399,400,401]
V-ATPase complex defects							
*ATP6V1A*	V-ATPase A subunit 1 (ATP6V1A)	AR	HCM	SD, RBBB	HF, LQTS	4	[14,405,406]
*ATP6V1E1*	ATPase subunit E (ATP6V1E1)	AR	HoRV, RVD, HCM	AD, MVR, TVR, PFO, SD, TI, AI, RHHS, TVS, PDA, MVP, RBBB	-	5	[14,405,407]

### 3.5. Disorders of Lysosomal Carbohydrate-Processing

Lysosomes are specialized organelles where complex components, such as glycans, proteins and other molecules, are chemically degraded in their fundamental components. Several lysosomal enzymes are involved in the finely organized degradation cascades of glycoconjugates and other complex molecules. The lysosomal catabolism of glycoproteins is an essential part of the cellular homeostasis of glycosylation. Once inside the lysosomes, glycoproteins are catabolized by acidic hydrolases. Here, the glycans are broken down and other posttranslational modifications such as sulfation, phosphorylation and esterification are removed as well [408]. All cellular glycoproteins continuously under turn-over and the degradationproducts (such as monosaccharides and amino acids) pass through the lysosomal membrane to be recycled by the cells. When this process is dysregulated, glycoproteins and other undegraded molecules accumulate in the lysosomes. Lysosomal storage diseases (LSDs) are inherited metabolic diseases which are caused by a deficiency of degradation enzymes, leading to abnormal build-up of a variety of toxic compounds [409]. Thus far, over 40 LSDs have been described and reported to mostly involve the skeleton, skin, brain and central nervous system [410]. Within this large family, we identified 15 LSDs that present with cardiac manifestations (Table 6).

#### 3.5.1. CTSA-LSD

Galactosialidosis (GSL), or Goldberg syndrome [OMIM#256540], is an AR LSD caused by homozygous or compound heterozygous mutations affecting the gene encoding cathepsin A (CTSA, also known as PPCA) on chromosome 20q13. CTSA is a carboxypeptidase exerting a protective and stabilizing function on β-galactosidase and neuraminidase. In fact, GSL is associated with a combined loss of function of these two enzymes and manifests in three different phenotypic subtypes: early infantile form, late infantile form and juvenile/adult form. Although the three forms share some clinical features (e.g., coarse facies, cherry red spots and foam cells in the bone marrow), they display some phenotypic differences and very different levels of severity and life expectancy. Cardiac involvement is usually reported in the infantile forms, which are also the most severe. Through our search, we identified 10 GLS patients, 8 infantile and 2 older in age (11 and 18 years old, respectively), described with cardiac involvement, specifically DC, heart murmur, SD (mainly thickened cardiac septa), altered LV wall thickness (with decreased ventricular function), CM, HF, VDL, arterial hypertension and presence of vacuolated myocardial fibers (Table 6, Supplementary Tables S2 and S3) [411,412,413,414,415,416,417,418,419,420]. Complex cyanotic congenital heart disease and situs inversus have also been reported in one of these cases [413].

#### 3.5.2. GBA1-LSD

Gaucher disease (GD) [OMIM:231000] is a rare AR subtype of the classical subacute/chronic neuronopathic GD caused by homozygous or compound heterozygous mutations affecting the glucosylceramidase-beta type 1 (*GBA1*, also known as acid beta-glucosidase) gene on chromosome 1q22. Within the lysosomal lumen, this enzyme hydrolyzes glucosylceramides, playing a central role in the glucosylation of cholesterol, degradation of complex lipids and turnover of the cellular membrane. This results in the accumulation of glucocerebrosides within cells such as immune cells, which can infiltrate several tissues such as heart, liver and muscles [421]. In addition to a severe perinatal–lethal form associated with ichthyosiform or collodion skin abnormalities or with nonimmune hydrops fetalis, three main types have been described [421]. GD type 1 is characterized by bone defects, hepatosplenomegaly, anemia, thrombocytopenia and lung disease without primary central nervous system disease. GD types 2 and 3 are instead predominantly characterized by primary neurologic disease and were once divided based on the onset age. In addition, GD type 3 can present with a cardiovascular form (GD3c) primarily associated with a specific *GBA1* mutation, namely p.D409H. Our search resulted in 141 patients with GD with cardiac involvement (mostly GD3c) [415,422,423,424,425,426,427,428,429,430,431,432,433,434,435,436,437,438,439,440,441,442,443,444,445,446,447,448,449,450,451,452,453,454,455,456,457,458,459,460,461,462,463,464]. The described cardiac manifestations include VC, valve thickening, ARe, MVR, AD, AVS, MVS, CMG, MF, LVH, LAE, RAE, TC, AF, BR, which escalates to HF (often congestive). Less frequently HCM, DCM, high–left ventricular wall thickness and intraventricular septum, AVB, recurrent syncope, palpitation and hemorrhagic pericarditis have also been reported (Table 6). Cardiovascular symptoms such as coronary artery disease and pulmonary hypertension have also often been described in GD patients.

#### 3.5.3. GLA-LSD

Fabry disease [OMIM:301500] is an X-linked inherited LSD caused by mutations on the α-galactosidase A (*GLA*) gene on chromosome Xq22. It has an estimated prevalence of approximately 1–5/10 000 live births. Genetic mutations on this gene induce deficient or absent activity of lysosomal enzyme GLA, which mediates glycosphingolipid catabolism. As a result, accumulation of globotriaoslyceramide (Gb3) and related glycosphingolipids have been detected in the plasma and cellular lysosomes of vessels, nerves, and other tissues [465,466]. Thus, GLA-LSD is a systemic disease that manifests with renal failure, cerebrovascular disease, small-fiber peripheral neuropathy, skin lesions and cardiac defects. Several thousand patients affected by GLA-LSD have been reported since its discovery in 1898, and many cardiac symptoms have been diagnosed as associated with this disease [467,468]. Cardiovascular manifestations predominantly include LVH (regional, apical, global), increased endomyocardial trabeculation, LV apical aneurysm, LVD, reduced strain (global and regional/segmental), RVH, RCM, reduced RV free wall strain, immune-mediate myocarditis, MF, congestive HF, increased filling pressure, DI, ARe, MVR, AVP, MVP, aorta and mitral valve thickening, LV outflow tract obstruction, reduced LA function (reservoir strain and strain rate), mild AD, coronary microvascular dysfunction, AT, BR, beat-to-beat variation in heart rate, chronotropic incompetence, AVB, short PR interval, increased arterial stiffness and elevated cardiac biomarkers (NT-proBNP, high-sensitivity troponins) [468]. Although cardiac involvement has been reported in patients affected by different mutations, one mutation affecting exon 5 of *GLA* gene, p.N215S (c.644A>G), has been confirmed to be causative of predominantly cardiac symptoms, mostly late-onset LVH, AT and conduction disturbances (Table 1) [469].

Many case reports and large cohort studies have been published thus far. For instance, one of the first large studies was published by Mehta et al., who investigated a cohort of 1453 GLA-LSD patients of which 798 (422 males and 376 females) presented cardiac symptoms such as LVH, LVM, valvular heart disease, conduction disturbances and hypertension (Table 6) [470]. In this cohort, cardiac complications represented the primary cause of death in both male (34%) and female (57%) patients.

Enzyme replacement therapy (ERT) is an established treatment for GLA LSD, with or without chaperone therapy (migalastat), which helps in preventing or delaying the progression of some complications such as LV mass increase; however, its effects on other cardiac symptoms such as LVH and on fibrotic damage are less clear [468].

#### 3.5.4. GLB1-LSD

Homozygous or compound heterozygous mutations of the gene encoding for beta-galactosidase-1 (*GLB1*) on chromosome 3p22.3 can originate two clinically distinct, albeit allelic, disorders: GM1-gangliosidosis type 1 and mucopolysacharidosis type IVB, also known by the eponym Morquio syndrome type B.

This enzyme hydrolyzes the terminal β-linked galactoside moieties from the glycans of gangliosides, glycosaminoglycans and other glycoconjugates within the lysosome, enabling their catabolism and recycling of monosaccharides. Loss of function of GLB1 leads to accumulation of gangliosides and sphingolipids in several organs, causing rapid psychomotor degeneration. Moreover, an alternative spliced transcript of the *GLB1* gene generates an elastin-binding protein (EBP), and some EBP mutations that compromise elastin fiber assembly have been associated with the development of cardiovascular symptoms [471,472].

GM1-gangliosidosis type 1 (GM1G1) [OMIM:230500] is an AR LSD with an estimated prevalence of about 1/100,000–200,000 live births. Clinically, GM1G1 is characterized by variable degrees of neurodegeneration and skeletal abnormalities, while cardiac symptoms are a less common occurrence, mostly associated with the infantile form and with specific *GLB1* mutations. The first clinical report of a GM1G1 case with cardiac involvement dates back to 1971 and describes an infantile patient with CM, incomplete RBBB, vacuolated and hypertrophied myofibers, thickened mitral valve leaflets with fibrous tissue and right coronary occlusion due to atherosclerotic plaque [473]. Later reports described 10 infantile patients with similar cardiac symptoms, predominantly DCM or HCM, LVH, CMG, systolic murmur and cases of death due to heart failure [474,475,476,477,478]. More recently, the spectrum of cardiac symptoms has been expanded by the description of 25 other patients presenting DCM or HCM, LVH, LVD, HSS and right interventricular conduction delay [472,479,480] (Table 6, Supplementary Tables S2 and S3).

Morquio syndrome type B [OMIM: 253010] is an AR LSD with a prevalence of 1/250,000–1,000,000 live births, with multiorgan involvement due to compromised keratan sulfate catabolism which results in intralysosomal accumulation in connective tissues and several other organs [481]. Clinically, this disease manifests with skeletal dysplasia, coarse facial features, corneal clouding, hearing disorder and cardiac (valvular) defects, usually without central nervous system involvement. Through our search, we identified eight patients with cardiac involvement, presenting MVS, AVS, VC and VF, MF, MVR, mitral dysplasia, ARe, DAR, SH, LVH, LV outflow tract obstruction and dyspnea with severe pulmonic autograft regurgitation [481,482,483] (Table 6, Supplementary Tables S2 and S3).

#### 3.5.5. HEXB-LSD

Sandhoff disease [OMIM:268800] is an AR LSD caused by mutations in the beta-hexosaminidase (*HEXB*) gene on chromosome 5q13. It has an estimated prevalence of 1–9/1,000,000 live births. This lysosomal hexosaminidase after activation catalyzes the degradation of the ganglioside GM2 and other molecules containing terminal N-acetyl hexosamines. In the case of HEXB loss of function, GM2 ganglioside is abnormally stored in several tissues, primarily neurons and peripheral nervous tissues (in fact, it is also classified as GM2-gangliosidosis type II). This translates to early blindness, progressive central nervous system degeneration, hepatosplenomegaly, macrocephaly and cherry red spots on the macula. Although less common in Sandhoff disease, nine infantile patients have been reported with cardiac manifestations [484,485,486]. All patients displayed MVP with MVR, ASD, VSD, CMG with LVD and HF [484,485,486,487,488,489,490] (Table 6, Supplementary Tables S2 and S3). A few patients also showed heart murmur and mild ARe from AVP, along with asymmetric hypertrophy of the interventricular septum without left ventricular outflow tract obstruction. These studies suggest that cardiac involvement in Sandhoff disease (though rare) can appear as an early sign during infancy.

#### 3.5.6. IDUA-LSD

Mucopolysaccharidosis type I is an AR LSD due to mutation of the gene encoding alpha-*L*-iduronidase (IDUA) on chromosome 4p16. IDUA hydrolyzes the terminal alpha-*L*-iduronic acid residues of two glycosaminoglycans (GAGs, also known as mucopolysaccharides), dermatan sulfate and heparan sulfate. From a clinical standpoint, MPS-I has been traditionally classified in subtypes based on the severity of the symptoms: the mild subtype called Scheie syndrome (MPS-IS) [OMIM: 607016], the moderate form called Hurler–Scheie syndrome (MPS-IH/S) [OMIM:607015] and the severe form called Hurler syndrome MPS-IH [OMIM:607014]. At present, however, MPS-I is considered a spectrum of disorders from attenuated to severe with many phenotypes in between [491]. Overall, it presents with neurological delay, coarse facial features, corneal clouding, hernias, hepatosplenomegaly, hearing impairment, skeletal malformations (e.g., scoliosis) and risk of infection, with pneumonia being one of the main causes of mortality along with cardiac failure [491]. Our systematic review results in 440 IDUA-deficient patients with cardiac manifestations, predominantly valvular disease (encompassing MVS, AVS, MVR, ARe), but also LVD, LVH leading to SI and DI, along with characteristic cardiovascular symptoms such coronary artery narrowing and/or occlusion [492,493,494,495,496,497,498,499,500,501,502,503,504,505,506,507,508,509,510,511,512,513,514,515,516,517,518,519,520,521,522,523,524,525,526,527,528,529,530,531,532,533,534,535,536,537,538,539,540,541,542,543,544,545,546,547,548,549,550,551]. In addition to standard pharmacological and surgical management, systemic therapies are also available for MPS-I (and other MPS types), namely hematopoietic stem cell transplantation (HSCT) and enzyme replacement therapy (ERT). Long-term metabolic correction by HSCT results in preservation of cardiac function and regression of cardiac, whereas long-term ERT may improve systolic ventricular function and resolution of LVD. Both therapies, though, did not show clear amelioration of valvular thickening, stenosis or regurgitation [491].

#### 3.5.7. IDS-LSD

Mucopolysaccharidosis type II [OMIM:309900], also known by the eponym Hunter syndrome, is an XLR LSD resulting from mutations of the gene encoding the enzyme iduronate 2-sulfatase (IDS), located on chromosome Xq28. IDS hydrolyzes the 2-sulfate groups of the *L*-iduronate 2-sulfate units of dermatan sulfate, heparan sulfate and heparin. When IDS is deficient, similarly to other MPS types, GAGs accumulate in the lysosomes in many tissues, leading to a multiorgan syndrome. Main clinical manifestations are represented by neurological decline, severe airway obstruction, skeletal deformities (e.g., scoliosis), hepatosplenomegaly, hearing impairment, cardiomyopathy and other valvulopathy. Unlike other MPS types, in MPS-II the lifespan can increase from the second decade up to the sixth decade, depending on the severity of the clinical presentation and the treatments received (e.g., ERT) [552]. Pneumonia and respiratory and cardiac failures are the most common causes of death in these patients [552,553]. Through our systematic review, we identified 742 MPS-II patients with cardiac symptoms, encompassing MVS, MVR, MI, AVS, ARe, AI, TI, DAR, ASD, AVB, CM/HCM, SH, AF, AT and cardiac murmur [497,509,512,540,544,553,554,555,556,557,558,559,560,561,562,563,564,565,566,567,568,569,570,571,572,573,574,575,576,577,578,579,580,581,582,583,584,585,586,587,588,589,590,591,592,593,594,595,596,597,598] (Table 6, Supplementary Tables S2 and S3). ERT and substrate reduction therapy in MPS-II patients prevent the accumulation of GAGs in peripheral tissues, thus reducing inflammation, risk of respiratory infections and coarseness of facial features and improving joint mobility, although no clear improvement in neurological deterioration and cardiac functionality have been documented [599]. HSCT instead appears to normalize hepatosplenomegaly and aortic valve deterioration [599].

#### 3.5.8. SGSH-LSD

Mucopolysaccharidosis type III subtype A [OMIM:252900], or Sanfilippo syndrome subtype A, is an AR LSD caused by homozygous or compound heterozygous mutations in the gene encoding *N*-sulfoglucosamine sulfohydrolase (*SGSH*) on chromosome 17q25. It is the most common subtype of MPS-III, with a prevalence of 1–1.62/100,000 newborns, depending on ethnicity and country [600,601]. The loss of function of this enzyme causes impaired degradation of GAGs, such as heparan sulfate and dermatan sulfate, which accumulate in the cell lysosomes of multiple tissues and compromise their functionality, leading to progressive deterioration of patients’ conditions. MPS-IIIA can be defined as an aggressive infantile-onset neurodegenerative disease traditionally presenting with intellectual disability, sleep disturbances, loss of ambulation, hepatomegaly, dysmorphism and early death [602]. Death usually occurs as a consequence of aggressive infections (mostly pneumonia) and of cardiac complications [603]. Through our search, we gathered (at least) 47 patients reported with CVD (MVS, MVR, AVS, ARe), BR, AT, VSD, asymmetric SH and CH and arrhythmogenic RVHCM, which can result in cardiac arrest and recommendation for heart transplant [532,589,604,605,606,607,608,609] (Table 6, Supplementary Tables S2 and S3). Additionally, we found eight studies with patients displaying cardiac involvement, but the subtype of Sanfilippo syndrome (A, B, C or D) was not specified, and thus they have not been included [512,540,544,556,564,591,600,610].

#### 3.5.9. NAGLU-LSD

Mucopolysaccharidosis type III subtype B [OMIM:252920], or Sanfilippo syndrome subtype B, is another AR LSD caused by compromised GAG degradation due to mutations affecting the *N*-alpha-acetylglucosaminidase (*NAGLU*) gene, located on chromosome 17q21. Its prevalence is estimated to be 0.5–1/100,000 live births [601]. Similarly to subtype A, NAGLU deficiency also results in lysosomal accumulation of partially degraded heparan sulfate in several tissues and manifests clinically with severe neurodegenerative symptoms and low life expectancy (second decade) [611]. However, subtype B is also characterized by severe skeletal defects and facial dysmorphism [611]. Our systematic search identified (at least) 39 MPS-IIIB patients described with cardiac symptoms, including CVD (MVS, MVR, AVS, ARe), to BR, AT, VSD, SH (asymmetric), CH and arrhythmogenic RVHCM, which in some cases resulted in cardiac arrest and/or recommendation for heart transplant [589,604,605,606,609,612,613,614] (Table 6, Supplementary Tables S2 and S3). As mentioned in the previous paragraph, we found eight additional studies with MPS_III patients described with cardiac involvement, but the subtype was not specified, thus these studies have been excluded (Supplementary Table S3) [512,540,544,556,564,591,600,610].

#### 3.5.10. HGSNAT-LSD and GNS-LSD

The rarest subtypes of mucopolysaccharidosis type III, namely subtype C [OMIM:252930] and D [OMIM:252940], are both hyper-rare AR LSDs with an unknown prevalence, caused by genetic mutations of two different genes: the *HGSNAT* gene (8p11.2-p11.1) and the *GNS* gene (12q14.3), respectively. The *HGSNAT* gene encodes heparan acetyl-CoA:alpha-glucosaminide *N*-acetyltransferase, while the *GNS* gene encodes *N*-acetylglucosamine-6-sulfatase. 

HGSNAT is a lysosomal acetyltransferase that acetylates the non-reducing terminal alpha-glucosamine residue of heparin and heparan sulfate in the presence of acetyl-CoA. When defective, intralysosomal GAG accumulation occurs and compromises the functionality of multiple organs, resulting in MPS-IIIC. This subtype has an estimated prevalence of 0.07–0.42/100,000 live births, depending on ethnicity and country [601,615]. MPS-IIIC associates with skeletal malformations and dysmorphic features, progressively delaying psychomotor development, hearing loss and sleep disturbances [616]. Through our systematic research, we gathered 10 patients who were also described with cardiac symptoms, such as CVD (MVS, MVR, AVS, ARe), LVH and SH; in at least one case, acute cardiac failure has also been reported [589,606,607,617] (Table 6, Supplementary Tables S2 and S3).

GNS is a glucosamine (*N*-acetyl)-6-sulfatase that hydrolyzes *N*-sulfate esters from heparin, heparan sulphate and keratan sulphate. This subtype has a prevalence estimated at around 0.10/100,000 [601] and is characterized by psychomotor delay, behavioral issues and dysmorphic features. Our systematic search produced two patients with MPS-IIID and cardiac involvement, specifically MR and SH (asymmetric) [609] (Table 6, Supplementary Tables S2 and S3). As mentioned in Section 3.5.8 and Section 3.5.9, eight additional studies with MPS-III patients with cardiac involvement were found, but in these studies the subtype was not specified, thus they have been excluded (Supplementary Table S3).

#### 3.5.11. GALNS-LSD

Mucopolysaccharidosis type IV is divided into two types: type A, also known by the eponym of Morquio syndrome A [OMIM:253000], and type B, or Morquio disease B [OMIM:253010, see Section 3.5.4], which originates from genetic defects on two different genes. Focusing on the former, MPS-IVA has a prevalence of 0.15–0.47/100,000 [618] and is caused by the absence of functional galactosamine-6-sulfatase, which results in the intralysosomal deposition and accumulation of several glycosylated substrates, primarily keratan sulfate and chondroitin sulfate. The spectrum of clinical features encompasses dwarfism, growth delay, skeletal malformations, dysplasia, cardiac symptoms such as cardiac valve thickening, CVD, MF, VF, MVR, ARe, TVR, MVS, AVS, TVS and CD [482,618,619,620,621,622] (Table 6). ERT and HSCT have both been applied and investigated in MAP-IVA patients and seems to at best normalize hypertrophy by reducing GAG accumulation and heart/valve fibrosis by controlling fibroblast infiltration, but in some cases, the valvulopathy worsened [623].

#### 3.5.12. ARSB-LSD

Mucopolysaccharidosis type VI [OMIM:253200], also known as Maroteaux–Lamy syndrome, is a progressive AR LSD with an estimated prevalence of 1/43,000–1,500,000 [624,625], caused by mutations affecting the *ARSB* gene on chromosome 5q14.1. This gene encodes arylsulfatase B (ARSB, or *N*-acetylgalactosamine 4-sulfatase), a sulfatase involved in the lysosomal catabolism of GAGs and which is mainly present in the liver and pancreas. When mutated, it results in the accumulation of partially degraded dermatan-sulphate and chondroitin-sulphate, in tissues and organs, which in turn causes an array of clinical manifestations that progressively worsen with age [625]. From a clinical standpoint, MPS-IV is divided into severe and attenuated forms, the latter further separated into cardiac type and osteoarticular type (although mixed cases can be found in the literature). Few specific mutations, predoinantly p.R152W, have been proved to specifically associated with the cardiac form [626]. Our search resulted in (at least) 520 MPS-IV patients presenting cardiac symptoms (in some comparative studies in which multiple MPS types were studied together, it was not always possible to determine the exact number of MPS-VI, and thus our count could be slightly underestimated; see Supplementary Table S3). The cardiac symptoms consisted primerily of cardiac valvular disease including VLD, valve thickening, DAR, MVP, AVP, TVP, MVR, ARe, TVR, MI, AI, TI, MVS, TVS and less frequently VSD (intraventricular septal hypertrophy), LHV, HCM and LVCR [497,512,532,540,544,556,564,570,574,579,589,590,591,596,625,626,627,628,629,630,631,632,633,634,635,636,637,638,639,640,641,642,643,644,645,646,647,648,649,650,651,652,653,654,655,656,657,658,659,660,661,662,663] (Table 6, Supplementary Tables S2 and S3). Cardiac involvement often escalated to congestive HF. ERT has been proven to stabilize and in some cases ameliorate hypertrophy-linked symptoms by reducing GAG accumulation; however, the beneficial effects on valvular defects are less clear and cases of worsened valvulopathies have also been reported [637,664].

#### 3.5.13. GUSB-LSD

Mucopolysaccharidosis type VII [OMIM:253220], also called Sly syndrome, is an ultra-rare AR LSD caused by mutations affecting the gene encoding beta-glucuronidase (GUSB) located on chromosome 7q11. The loss of function of GUSB results in the lysosomal accumulation of three main GAGs, dermatan sulfate, heparan sulfate and chondroitin sulfate, leading to tissue hypertrophy and function disruption of many organs [665]. MPS-VII is clinically characterized by degenerative neurological symptoms, severe skeletal malformations (mostly thoracic deformity, scoliosis, and hip dysplasia), facial dysmorphism, hepatosplenomegaly, susceptibility to infections of the ear and respiratory tract, pulmonary complications and cardiac defects [665]. Our search resulted in 46 MPS-VII patients with cardiac involvement consisting of progressive CVD, ARe, AVS, AI, DAR, MVR, MVS, MI, TVR, MF, LVD, LVH, CH, early repolarization and T wave inversion and, in several cases, congestive HF [540,564,590,654,666,667,668,669,670,671,672,673,674,675] (Table 6, Supplementary Tables S2 and S3). ERT in MPS-VII seems to stabilize some of the cardiac symptoms (although worsening cases have also been reported), while the effects on these symptoms due to HSCT are still under scrutiny in few clinical trials [676].

#### 3.5.14. ARSK-LSD

Mucopolysaccharidosis type X [OMIM:619698] is a very recently described type of AR LSD caused by homozygous or compound heterozygous mutations of the *ARSK* gene on chromosome 5q15. This gene encodes arylsulfatase K, which is responsible for the hydrolysis of *N*-sulfate esters from sulfated multiple substrates such as GAGs, steroids, carbohydrates, proteoglycans and glycolipids. When loss of function occurs, the catabolism of GAGs (e.g. heparan sulfate, chondroitin sulfate, dermatan sulfate) and other substrates is compromised, and they accumulate in peripheral tissues disrupting their functionality [677]. Thus far, only 4 cases from two different families have been reported with this disease. Of these, 2 patients (siblings) were described with cardiac complications, in the form of AVS, MVS, ARe, MVR, thickened ends of aortic cusps, LVH and systolic murmur [678] (Table 6, Supplementary Tables S2 and S3).

**Table 6:** Lysosomal storage diseases for which patients displaying cardiac manifestations have been reported (abbreviations described in Table 1).

Affected Gene	Affected Protein	Inheritance	Heart Defects & Manifestations			No. Patients Identified by Our Search	Ref.
			Cardiomyopathies	Structural Defects	Arrhythmogenic Disorders		
*CTSA*	Cathepsin A (CTSA)	AR	CM, LVRWMA	DC, SD, VLD	HF	10	[411,412,413,414,415,416,417,418,419,420]
*GBA1*	Glucocerebrosidase (GBA)	AR	LVH, HCM, DCM	VC, ARe, MVR, CMG, MF, AVS, MVS, LAE, RAE, AVB	AF, TC, BR, HF	141	[415,422,423,424,425,426,427,428,429,430,431,432,433,434,435,436,437,438,439,440,441,442,443,444,445,446,447,448,449,450,451,452,453,454,455,456,457,458,459,460,461,462,463,464]
*GLA*	α-galactosidase (GLA)	XL	LVH, LVD, RVH, RCM	ARe, MVR, AD, MF, DI, MVP, AVP, AVB	AT, BR	>300	[467,468,469,470] * selected
*GLB1*	β-galactosidase (GLB1)	AR (GM1 gangliosidosis)	DCM, LVH, LVD, HCM	RBBB, HSS	HF	25	[472,473,474,475,476,477,478,479,480]
*GLB1*	β-galactosidase (GLB1)	AR (mucopolysaccharidosis)	LVH, SH	ARe, DAR, MVR, MVS, AVS, MF, VF	-	8	[481,482,483]
*HEXB*	β-hexosaminidase B (HEXB)	AR	HCM, LVD	CMG, ARe, MVR, MVP, MVS, AVP, VLD	HF	9	[484,485,486,487,488,489,490]
*IDUA*	α-*L*-iduronidase (IDUA)	AR	LVD, LVH	ARe, MVR, MVS, AVS, SI, DI	HF	440	[492,493,494,495,496,497,498,499,500,501,502,503,504,505,506,507,508,509,510,511,512,513,514,515,516,517,518,519,520,521,522,523,524,525,526,527,528,529,530,531,532,533,534,535,536,537,538,539,540,541,542,543,544,545,546,547,548,549,550,551]
*IDS*	2-sulfatase (IDS)	XLR	HCM, SH	Are, MVR, DAR, MVS, MI, AVS, AI, TI	AF, AT	742	[497,509,512,540,544,553,554,555,556,557,558,559,560,561,562,563,564,565,566,567,568,569,570,571,572,573,574,575,576,577,578,579,580,581,582,583,584,585,586,587,588,589,590,591,592,593,594,595,596,597,598]
*SGSH*	*N*-sulfoglucosamine sulfohydrolase	AD	SH, CH, RVHCM	CVD, ARe, MVR, MVS, AVS, VSD	BR, AT, HF	47	[532,589,604,605,606,607,608,609]
*NAGLU*	*N*-α-acetylglucosaminidase (NAGLU)	AD	SH, CH, RVHCM	ARe, MVR, CVD, MVS, AVS, VSD	BR, AT	39	[589,604,605,606,609,612,613,614]
*HGSNAT*	heparan acetyl-CoA:alpha-glucosaminide *N*-acetyltransferase (HGSNAT)	AD	SH, CH RVHCM	ARe, MVR, CVD, MVS, AVS, VSD	BR, AT, HF	10	[589,606,607,617]
*GNS*	glucosamine (*N*-acetyl)-6-sulfatase (GNS)	AD	SH	MVR	-	2	[609]
*GALNS*	galactosamine (*N*-acetyl)-6-sulfatase (GALNS)	AD	CD	ARe, MVR, TVR, CVD, MF, VF, AVS, MVS, TVS	-	>300	[482,618,619,620,621,622] *selected
*ARSB*	*N*-acetylgalactosamine 4-sulfatase (ARSB)	AD	VSD, SH, HCM	DAR, MVR, ARe, TVR, VLD, MVP, AVP, TVP, AI, MI, MVS, TVS, LVCR	HF	520	[497,512,532,540,544,556,564,570,574,579,589,590,591,596,625,626,627,628,629,630,631,632,633,634,635,636,637,638,639,640,641,642,643,644,645,646,647,648,649,650,651,652,653,654,655,656,657,658,659,660,661,662,663]
*GUSB*	β-glucuronidase (GUSB)	AD	LVD, LVH, CH	ARe, DAR, MVR, TVR, CVD, AVI, AVS, MVS, MI, MF	HF	46	[540,564,590,654,666,667,668,669,670,671,672,673,674,675]
*ARSK*	arylsulfatase K (ARSK)	AD	LVH	ARe, MVR, AVS, MVS	=	1	[678]

* For GAA deficiency our search produced over 300 entries, thus only some meaningful articles were selected.

## 4. Conclusions

**Figure 2:**
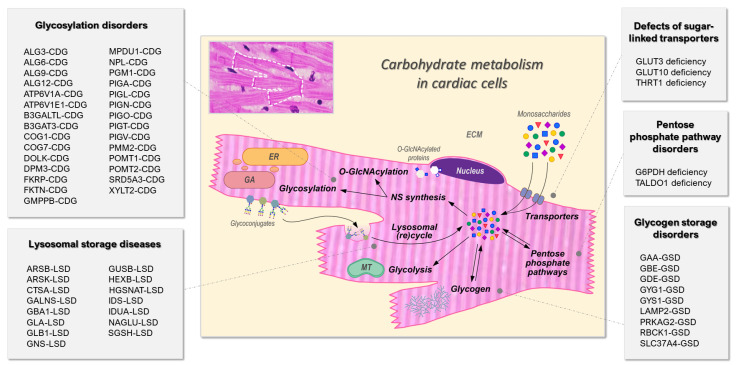
Summary of the inherited disorders of carbohydrate metabolism associated to cardiac manifestations, contextualized with the main carbohydrate metabolic pathways in cardiac cells. Abbreviations: CDG, congenital disorder of glycosylation; ECM, extracellular matrix (yellow); ER, endoplasmic reticulum (orange); GA, Golgi apparatus (red); GSD, glycogen storage disorder; MT, mitochondrion (green). Other symbols: fucose molecules, red triangles; galactose molecules, yellow circles; glucose molecules, blue circles; GlcNAc molecules, blue squares; mannose molecules, green circles; sialic acid molecules, purple diamonds; transporters, grey ovals; cell nucleus colored in purple; cytoplasm colored in pink.

The heart is the organ responsible for providing and maintaining the blood supply to all tissues of the body. Its muscular component, called myocardium, is formed by highly specialized contractile cells, extremely resistant to fatigue and characterized by unparalleled metabolic plasticity. To meet the high energy demand required by the contractile machinery of these cells, the biochemistry of the human myocardium has evolved to ensure constant energy production from a variety of substrates, mainly lipids and carbohydrates [679,680]. Carbohydrates do not only serve as substrates for energy production, but also as precursors for energy storage (via glycogen), macromolecule synthesis (via ribose 5-phosphate) and maturation (via glycosylation). In our review of the literature, we identified 58 congenital metabolic disorders affecting carbohydrate metabolism which are associated with cardiomyopathies, arrhythmogenic disorders and/or structural defects, variable in severity and presentation (Figure 2). Although for some of the diseases discussed hereinabove the cardiac complications may be secondary to other mechanism (such as dysfunctional autophagy in LAMP2 deficiency), our results stress the pivotal role of carbohydrate-linked mechanisms in the formation, development and contractile function of the heart.

With this systematic review, on one hand, we intend to raise awareness among clinicians about the potential association between rare IMDs and cardiac symptoms, as aid for diagnostics and therapeutics. On the other hand, by systematically gathering information on the enzymatic deficiencies that lead to cardiac manifestations, we aim to provide relevant knowledge on the molecular mechanisms underlying specific processes involved in cardiac development and functionality, which could be targeted for pharmacologic therapy and metabolic interventions.

## Figures and Tables

**Table 1:** Classification of cardiomyopathies, arrhythmogenic disorders and structural defects used in the systematic search.

Arrhythmogenic Disorders		Cardiomyopathies		Cardiac and Valvular Defects	
AT	Arrhythmia	ASH	Atrial septal hypertrophy	AD	Aortic dilation
AF	Atrial fibrillation	BVD	Biventricular dilation	AC	Aortic coarctation
BR	Bradycardia	BVH	Biventricular hypertrophy	AF	Atrial fibrosis
HF	Heart failure	CD	Cardiac dilation	AI	Aortic insufficiency
LQTS	Long-QT syndromes	CH	Cardiac hypertrophy	ARe	Aortic regurgitation
TC	Tachycardia	DI	Diastolic impairment (or dysfunction)	ASD	Atrial septal defects
VF	Ventricular fibrillation	DCM	Dilated cardiomyopathy	AVB	Atrioventricular block
		HCM	Hypertrophic cardiomyopathy	AVD	Aortic valve dysplasia
		HOCM	Hypertrophic obstructive cardiomyopathy	AVP	Atrial valve prolapse
		HoRV	Hypoplastic right ventricle	AVS	Aortic valve stenosis
		HoLV	Hypoplastic left ventricle	BAV	Bicuspid aortic valve
		IC	Ischemic cardiomyopathy	BPV	Bicuscpid pulmonary valve
		IOC	Iron overload cardiomyopathy	CMG	Cardiomegaly
		LCDM	Dilated ventricular cardiomyopathy	CVD	Cardiac valve defects
		LHCM	Left HCM	DAR	Dilated aortic root
		LVHCM	Left ventricular HCM	DC	Dextrocardia
		LVCO	Left ventricular cavity obliteration	DOV	Double outlet ventricle
		LVD	Left ventricular dilation	EA	Ebstein anomaly
		LVH	Left ventricular hypertrophy	ECD	Endocardial cushion defect
		LVRWMA	Left ventricular regional wall motion abnormality *	HSS	Hypertrophic subaortic stenosis
		NCM	Non-compaction cardiomyopathy	LAE	Left atrial enlargement
		RAD	Right atrial dilation	LBBB	Left bundle branch block
		RCM	Restrictive cardiomyopathy	LVCR	Left ventricular concentric remodeling
		RHCM	Right HCM	MF	Myocardial fibrosis
		RVD	Right ventricular dilation	MI	Mitral insuffiency
		RVDe	Right ventricular defect	MVP	Mitral valve prolapse
		RVH	Right ventricular hypertrophy	MVR	Mitral valve regurgitation
		RVHCM	Right ventricular HCM	MVS	Mitral valve stenosis
		SH	Septal hypertrophy	OA	Overriding aorta
		SI	Systolic impairment (or dysfunction)	PDA	Patent (persistent) ductus arteriosus
		VD	Ventricular dysfunction	PFO	Patent (persistent) foramen ovale
		VEFR	Ventricular ejection fraction reduced	PMV	Parachute mitral valve
		VH	Ventricular hypertrophy	PPS	Peripheral pulmonary stenosis
				PVSD	Perimembranous ventricular septal defect
				RAE	Right atrial enlargement
				RBBB	Right bundle branch block (associated with structure)
				RDA	Right-descending aorta
				RHHS	Right hypoplastic heart syndrome
				SAI	Small aortic isthmus
				SD	Septal defect
				SCA	Single coronary artery
				TGA	Transposition of the great arteries
				TI	Tricuspid insufficiency
				ToF	Tetralogy of Fallot (VSD, aortic dextroposition, PPS, RVH)
				TVR	Tricuspid valve regurgitation
				TVS	Tricuspid valve stenosis
				VC	Valvular calcification
				VF	Ventricular fibrosis
				VSD	Ventricular septal defect

* Left ventricular regional wall motion abnormality is defined as a LV segment in which the systolic motion score is below normal.

## Supplementary Materials

The following supporting information can be downloaded at: https://www.mdpi.com/article/10.3390/ijms24108632/s1.
